# Enterotoxin tilimycin from gut-resident *Klebsiella* promotes mutational evolution and antibiotic resistance in mice

**DOI:** 10.1038/s41564-022-01260-3

**Published:** 2022-10-26

**Authors:** Sabine Kienesberger, Amar Cosic, Maksym Kitsera, Sandra Raffl, Marlene Hiesinger, Eva Leitner, Bettina Halwachs, Gregor Gorkiewicz, Ronald A. Glabonjat, Georg Raber, Christian Lembacher-Fadum, Rolf Breinbauer, Stefan Schild, Ellen L. Zechner

**Affiliations:** 1 Institute of Molecular Biosciences, University of Graz, Graz, Austria; 2 BioTechMed-Graz, Graz, Austria; 3 Field of Excellence BioHealth, University of Graz, Graz, Austria; 4 Diagnostic and Research Institute of Hygiene, Microbiology and Environmental Medicine, Medical University of Graz, Graz, Austria; 5 Institute of Pharmaceutical Sciences, University of Graz, Graz, Austria; 6 Diagnostic and Research Institute of Pathology, Medical University of Graz, Graz, Austria; 7 Institute of Chemistry, University of Graz, NAWI Graz, Graz, Austria; 8 Institute of Organic Chemistry, Graz University of Technology, Graz, Austria

**Keywords:** Microbial ecology, Mechanism of action, Mutation, Evolutionary biology, Pathogens

## Abstract

*Klebsiella* spp. that secrete the DNA-alkylating enterotoxin tilimycin colonize the human intestinal tract. Numbers of toxigenic bacteria increase during antibiotic use, and the resulting accumulation of tilimycin in the intestinal lumen damages the epithelium via genetic instability and apoptosis. Here we examine the impact of this genotoxin on the gut ecosystem. 16S rRNA sequencing of faecal samples from mice colonized with *Klebsiella oxytoca* strains and mechanistic analyses show that tilimycin is a pro-mutagenic antibiotic affecting multiple phyla. Transient synthesis of tilimycin in the murine gut antagonized niche competitors, reduced microbial richness and altered taxonomic composition of the microbiota both during and following exposure. Moreover, tilimycin secretion increased rates of mutagenesis in co-resident opportunistic pathogens such as *Klebsiella pneumoniae* and *Escherichia coli*, as shown by de novo acquisition of antibiotic resistance. We conclude that tilimycin is a bacterial mutagen, and flares of genotoxic *Klebsiella* have the potential to drive the emergence of resistance, destabilize the gut microbiota and shape its evolutionary trajectory.

## Abstract (web summary)

Production of the enterotoxin tilimycin by gut-resident *Klebsiella* species can alter gut microbiota composition, induce mutational evolution and drive the emergence of antibiotic resistance in mice.

## Main

The human gut microbiota has vast functional potential^1,2^. Tens of thousands of microbially produced small molecules are released in the intestine^3–5^. The majority of these small molecules remain uncharacterized, and their impact on ecosystem function and human health is unknown^2,6–8^. Understanding the role of metabolites as regulators of interactions between microbes and host cellular responses is a major research area^2,9–11^, yet in most cases the molecular mechanisms underlying these functional networks remain elusive.

Tilimycin (TM) and tilivalline (TV) are small-molecule cytotoxins produced by the non-ribosomal peptide synthetase *til* pathway^12^. In the human gut ecosystem, the *til* gene cluster is restricted to members of the *Klebsiella oxytoca* species complex, particularly *K. oxytoca* and *K. grimontii*^13,14^. Bacteria belonging to this complex are early colonizers of the infant gut^15,16^; a feature they share^17^ with *Escherichia coli* phylogroups producing the genotoxic secondary metabolite colibactin^18^. Unlike colibactin, which is transferred by a cell-contact-dependent mechanism^2,9,19^ *til* cytotoxins are secreted to the external milieu in chemically stable forms that accumulate in the intestinal lumen^20,21^. Under conditions of antibiotic-induced dysbiosis, *til*^+^ bacteria expand and the burst of cytotoxins they release induces antibiotic-associated haemorrhagic colitis (AAHC) in children and adults^22,23^. High carriage rates have been reported for newborns delivered by caesarian section^15^, and cytotoxic strains are associated with necrotizing enterocolitis in premature infants^24^. TM’s enterotoxicity is due to adduct formation with guanines in host DNA^25^. DNA alkylation leads to strand breakage, genome instability and cell death^20^. By contrast, biochemical experiments have shown that TV exerts a stabilizing effect on microtubules, enhancing both nucleation and elongation phases of polymerization. The resulting inhibition of microtubule disassembly impedes mitosis and induces apoptosis^20^.

Consistent with their distinct molecular targets, limited in vitro data note antibiotic activity for TM, but no anti-bacterial effects of TV are known^20^. As bacteria in the gut must compete for limited nutrients and physical space, many bacteria have developed killing mechanisms to compete with members of the same species and others that vie for the same nutrients and locations^26^. Some involve contact-dependent transport of negative effectors from donor to recipient cells^19,27,28^. Others release noxious small molecules such as bacteriocins^2,6^. In either case, efficacy can be limited by narrow activity ranges or the need for target bacteria to be in close physical proximity. In contrast, TM’s small size (234.3 Da) and stable bioactive form may enable it to diffuse spatially and exert inhibitory effects locally and at a distance including also organisms that are not direct competitors. It is reasonable to propose, therefore, that luminal accumulation of TM not only affects host cells directly but also alters the structure and functions of the gut microbiota.

In this Article, we ask how an active *til* pathway impacts taxonomic composition of the gut microbiota and interrogate TM’s anti-bacterial mode of action, focusing on its genotoxic potential. Our rationale followed three lines of reasoning. First, repair of TM-alkylated DNA in bacteria should rely on error-prone DNA repair systems and thus directly induce mutations. Second, low doses of antibiotics can elevate mutation rates in some bacterial species. This adaptive response can occur independently of the drug’s cellular target as a byproduct of oxidative metabolism and the SOS and RpoS-regulated general stress responses^29^. In either case, TM should invoke transient hypermutation in exposed bacteria. Third, induction of hypermutable phenotypes on an inter-species level has the potential to generate a wide range of genetic, metabolic and functional variation. We demonstrate that genotoxic *K. oxytoca* alter the composition of the host microbiota during periods of toxin production as well as the succession communities that evolve after toxin exposure. We further show mutational resistance emergence in colonizing ESKAPE pathogens as one clinically important example of toxin-induced gain of function.

## Results

### TM depletes richness and shifts gut taxonomic composition

**Fig. 1:**
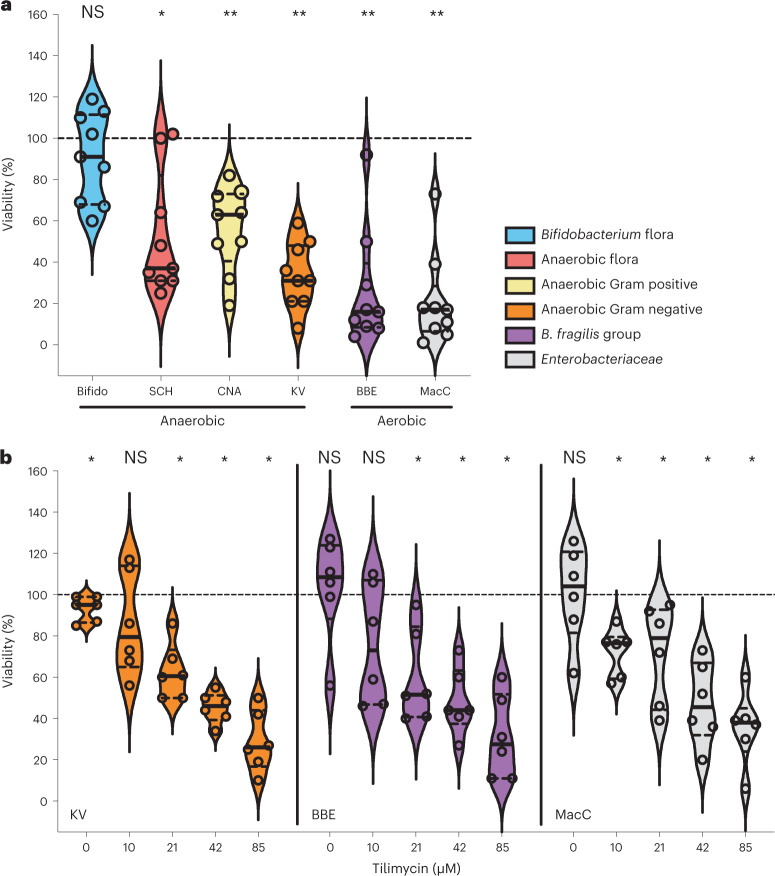
Anti-bacterial activity of TM against human faecal bacteria. **a**, Stool samples from healthy donors (*n* = 9) were exposed to 170 µM TM or solvent (EtOH), and the indicated bacterial groups were selectively enriched on differential media. **b**, Treatment of stool samples (*n* = 6) was repeated with a range of lower TM concentrations (1–85 µM) or EtOH before selection for the indicated bacterial groups. In **a** and **b**, violin plots (median, quartiles and all points) show percentage of survival relative to EtOH control for each group. Two-sided Wilcoxon signed-rank test towards 100 compared EtOH and TM treatment for the respective pair and group. *P = 0.05, **P = 0.01. NS, not statistically significant. Source data

**Extended Data Fig. 1:**
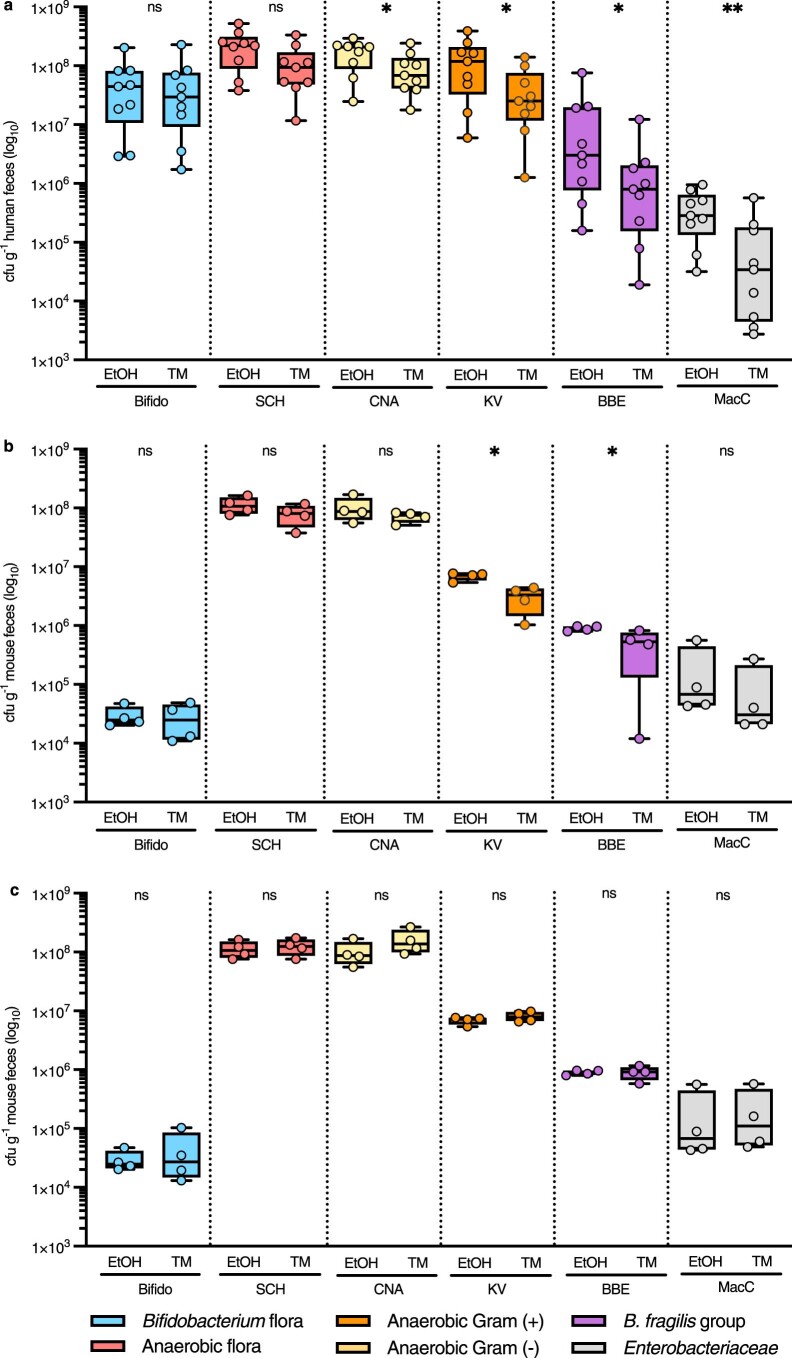
Cfu obtained for human and mouse fecal samples post-metabolite exposure. **(a-c)** Boxplots (center lines represent medians, box limits represent upper and lower quartiles, whiskers span min. to max. values, all points shown) compare **(a)** bacterial cell counts from human stool samples (n = 9) after treatment with EtOH or 170 µM TM on differential media, or **(b, c)** compare bacterial cell counts in mouse feces (n = 4) after EtOH treatment with cell counts after **(b)** 170 µM TM or **(c)** 17 µM TV treatment. **(a-c)** Statistical significance was evaluated by one-tailed Mann-Whitney test. *p = 0.05, **p = 0.01, (ns) not statistically significant. *Bifidobacterium* agar (Bifido); Schaedler agar (SCH); Schaedler colistin-nalidixic acid agar (CNA); Schaedler kanamycin-vancomycin agar (KV); Bacteroides Bile Esculin agar (BBE); MacConkey agar (MacC). Source data

We first asked whether the anti-bacterial activity ascribed to TM against single species in vitro may be relevant for the structure and function of the human gut microbiota. Faecal samples from healthy subjects were exposed briefly to a concentration of TM we typically measure in mouse models of antibiotic-induced dysbiosis^20^. Surviving cells were then cultured anaerobically on differential media to select specific groups of bacteria (Fig. 1a and Extended Data Fig. 1a). Treatment with TM compared with the ethanol solvent control resulted in significantly fewer colony-forming units (CFU) g^−1^ faeces on the majority of selection media. Strong post-exposure inhibition of Enterobacteriaceae was also observed. We conclude that TM exerts broad-spectrum bactericidal activity against culturable Gram-positive and Gram-negative members of the human faecal microbiota.

Few quantitative data are available for patients with AAHC, but faecal densities of toxigenic *K. oxytoca* and *K. grimontii* ranging from 10^7^ to 10^8^ CFU g^−1^ have been reported^30,31^. The analyte levels from just one patient stool sample known to contain 10^7^ viable *K. oxytoca* cells g^−1^ has been published (1.1 ± 0.1 nmol g^−1^ TM and 8 ± 1 pmol g^−1^ TV (wet weight))^32^. On the basis of these limited data, it is conceivable that expansion of TM-producing bacteria may be generally less pronounced in patients taking antibiotics than in mouse models (10^9^–10^10^ CFU g^−1^). We therefore lowered the range of TM concentrations tested to match these rare reports. The same stool samples of healthy donors were treated with diluted TM and plated to select the most TM-sensitive bacterial groups (Fig. 1b). Despite inter-individual variation, the genotoxin mediated significant inhibition of the human samples across this range. Moreover, Gram-negative anaerobes and Enterobacteriaceae were suppressed by the lowest 1–10 µM levels that can be extrapolated from the available patient data. TM is thus effective against gut microbes within a quantitative range relevant for human carriers.

To establish whether TM exerts anti-bacterial activity in the gut ecosystem, we designed a mouse infection experiment using a mutant strain of *K. oxytoca*. Loss of biosynthesis gene *npsB* eliminates production of TM and the second product of the pathway, TV, as this is formed by spontaneous condensation of TM and indole^12^. TM-deficient mutants of *K. oxytoca* are thus, by default, also TV deficient. TV lacked anti-bacterial activity in previous tests^20^. Nonetheless, because we cannot eliminate just TM using genetics, we treated samples of murine faecal bacteria with each metabolite separately. Suppression of the murine microbiota was observed with TM (Extended Data Fig. 1b), but TV exposure had no effect (Extended Data Fig. 1c). Therefore, we refer solely to the anti-bacterial activity of TM throughout this report.

**Fig. 2:**
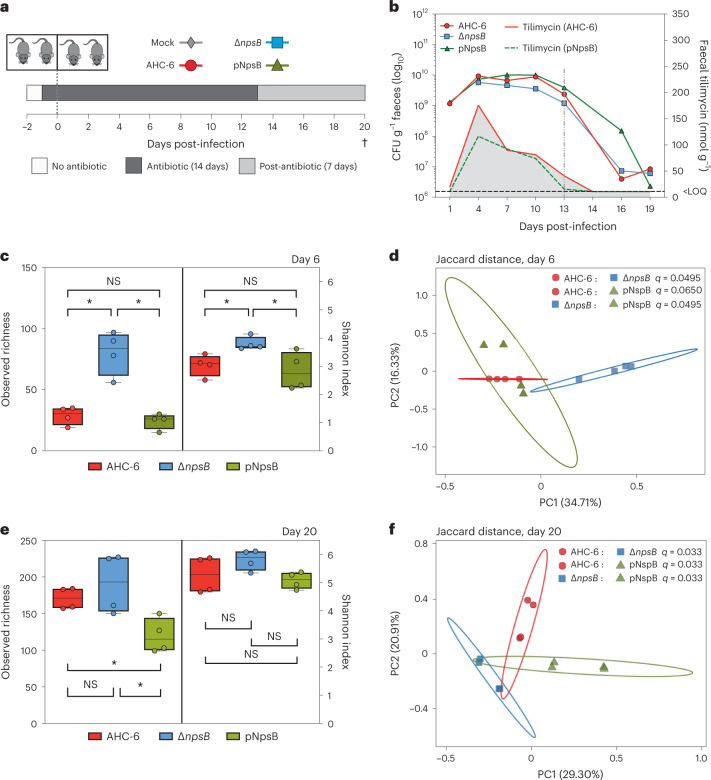
Genotoxic *K. oxytoca* reduce richness of the murine gut microbiota. **a**, Longitudinal study design. Cohorts received Curam 24 h before gavage with sterile broth (mock); toxigenic *K. oxytoca* AHC-6, TM/TV deficient Δ*npsB;* or Δ*npsB* complemented with *npsB* in trans (pNpsB). Curam was withdrawn 13 days post-infection. Mice were killed on day 20 after 7 days post-antibiotic recovery. **b**, Quantification of faecal TM and cell counts of *K. oxytoca* obtained from infected mice (*n* = 4 per group). Shown is geometric mean of CFU g^−1^ faeces and mean average concentrations of TM (grey shading) for each group over time (AHC-6, red line; pNpsB, dotted green line). LOQ denotes limit of quantification for TM. Dotted line marks end of Curam treatment. Data for individual mice are presented in Extended Data Fig. 2b. **c**,**e**, Comparison of alpha-diversity indices in faeces at day 6 (**c**) and in caecal content at day 20 (**e**) post-infection. Box plots (centre lines represent medians, box limits represent upper and lower quartiles, whiskers span minimum to maximum values, all points shown) of observed sequence variants (observed richness, left) and Shannon indices (right) for all treatments. Statistical significance by Kruskal–Wallis (all groups) followed by Kruskal–Wallis (pairwise) analysis. *q < 0.05, after Benjamini–Hochberg FDR correction. **d**,**f**, 2D-PCoA plots of Jaccard distance (beta diversity) on day 6 (**d**) and day 20 (**f**) post-infection. Statistical significance was evaluated by pairwise PERMANOVA with Benjamini–Hochberg FDR correction (Extended Data Fig. 2), *q < 0.05. Ellipses represent the 95% confidence interval. Source data

**Extended Data Fig. 2:**
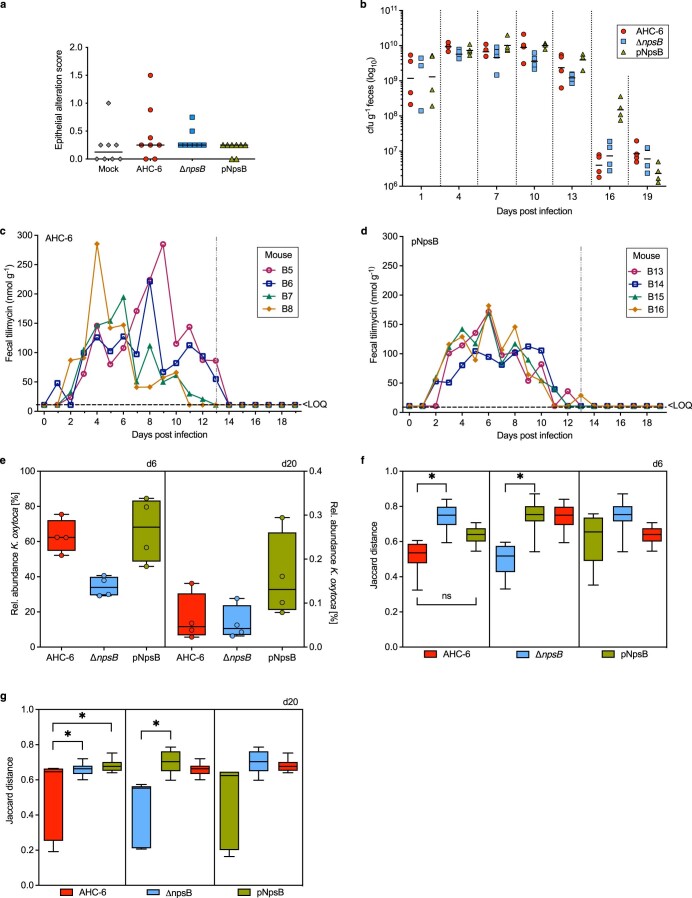
Variation in *K. oxytoca* population size and TM impact on beta-diversity over time. **(a)** Histopathologic evaluation of murine intestinal tissue. Epithelial cell alteration scores at day 4 post-gavage with sterile broth (Mock), *K. oxytoca* AHC-6, Δ*npsB*, or pNpsB (n = 8 mice/group). No significant difference was detected between groups (Kruskal-Wallis test). **(b)** Fecal load of *K. oxytoca* for mice colonized with AHC-6, Δ*npsB*, pNpsB (n = 4 mice/group) during and following Curam treatment (after day 13). Shown are individual values and the geometric mean (bars) of cfu g^−1^ feces. **(c, d)** Daily fecal TM levels per mouse (n = 4) for **(c)** AHC-6 and **(d)** pNpsB complementation cohorts. Dotted line marks withdrawal of Curam. LOQ denotes limit of quantification for TM. **(e)** Boxplots of relative abundance (based on 16 S rRNA sequences) for *K. oxytoca* AHC-6, Δ*npsB*, and pNpsB on day 6 and day 20 post-infection (n = 4 mice/group). **(f, g)** Boxplots of pairwise comparisons of Jaccard distances between the treatment groups (n = 4 mice/group) for **(f)** day 6 and **(g)** day 20 post-infection. **(e,f,g)** Boxplot center lines represent medians, box limits represent upper and lower quartiles, whiskers span min. to max. values, all points shown. **(f, g)** Statistical significance was evaluated by two-tailed pairwise PERMANOVA with Benjamini-Hochberg FDR correction. *q < 0.05. Source data

In the murine model (Fig. 2a), we recapitulated the dysbiosis that typically precedes *K. oxytoca* overgrowth in patients taking ß-lactams by adding Curam (amoxicillin/clavulanate) to drinking water. Compared with previous studies^33^, omission of an anti-inflammatory drug, which acts as an aetiological co-factor in AAHC, minimized epithelial damage (Extended Data Fig. 2a). Mice were inoculated orally 1 day post-Curam with *K. oxytoca* AHC-6, its Δ*npsB* mutant, or this mutant complemented with *npsB* in *trans*. Density of *K. oxytoca* and the quantity of TM in faeces was monitored daily (Fig. 2b). Values for individual animals are presented in Extended Data Fig. 2b–d. Measurable TM was produced rapidly, reaching maxima between 4 and 10 days, then declining until day 14 (Fig. 2b and Extended Data Fig. 2c,d). After this time *K. oxytoca* persisted in moderate abundance (Fig. 2b and Extended Data Fig. 2b); thus, it is possible that TM was also present in vivo, albeit under our level of quantitation. TV was detected in the *til* + infection groups but remained under the limit of quantitation throughout the course of infection. 16S rRNA gene sequence analyses of the faecal microbiome at day 6 (Fig. 2c) revealed significantly less richness in both groups carrying genotoxic strains (AHC-6 and complementation) compared with animals colonized with non-producers (Δ*npsB*). Moreover, those taxa present in the absence of TM were more evenly distributed than the communities observed in both toxin-producing cohorts (Shannon index; Fig. 2c). The relative abundance of *K. oxytoca* was generally high in each infection group at day 6 (Extended Data Fig. 2e) but notably lower for non-producers. Principal coordinate analysis (PCoA) based on Jaccard’s distance revealed that the composition of the communities formed in the presence of genotoxic strains was completely separated from communities free of TM (Δ*npsB*) but did not differ significantly from each other (Fig. 2d and Extended Data Fig. 2f).

Since expansion of TM producers is coupled to antibiotic disturbance, we next asked whether previous TM exposure would also shape the process of bacterial community succession during antibiotic recovery. Curam was discontinued on day 13 to reflect a typical course of patient therapy. Caecal content was sampled at day 20 at the study endpoint. During the 7 day post-Curam recovery time, *K. oxytoca* was suppressed to a relative abundance of less than 1% (Extended Data Fig. 2e). Analysis of alpha-diversity indexes (Fig. 2e) revealed increased richness in all post-antibiotic successions relative to day 6 (Fig. 2c). The only significant variation between observed richness was noted for the complementation group compared with AHC-6 and Δ*npsB* (Fig. 2e). However, the Shannon indices increased in the succession, leaving no significant difference between groups (Fig. 2e). Analysis of beta diversity, by contrast, showed that the phylogeny of each successive community diverged significantly from the others (Fig. 2f and Extended Data Fig. 2g). The results of this initial experiment thus show that, in the dysbiotic murine intestine, production of TM substantially restricts richness and evenness of the co-resident community while driving phylogenetic change. Moreover, the communities that evolve following antibiotic challenge differ significantly.

### Genotoxin alters bacterial succession post-exposure

**Fig. 3:**
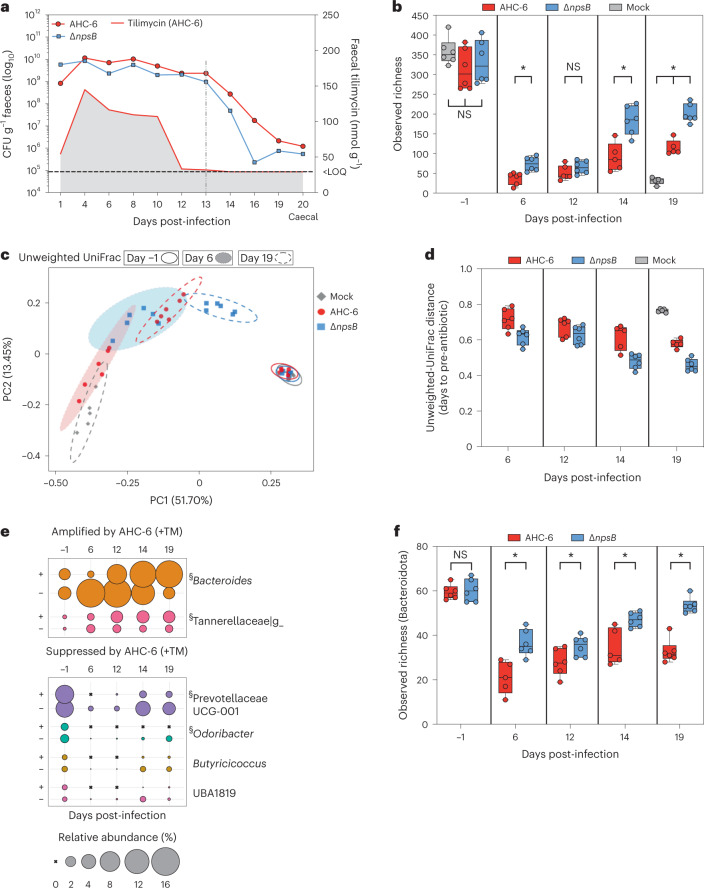
Variance of microbial diversity and composition during TM exposure and in the subsequent community succession. **a**, Quantification of TM and *K. oxytoca* in faeces and caecal content (day 20) of mice colonized with AHC-6, Δ*npsB* (*n* = 6 mice per group) or mock control animals (*n* = 6, no *K. oxytoca* or TM detected). Shown are the geometric means of CFU g^−1^ faeces, and red line with grey shading designates average TM concentrations (mean) for AHC-6 mice over time. Dotted line marks withdrawal of Curam 13 days post-infection. LOQ denotes limit of quantification for TM. Data for individual mice are shown in Extended Data Fig. 3a,b. **b**, Box plots compare median values of observed sequence variants (observed richness) before infection and over time post-infection for each treatment group (*n* = 6 mice per group except day 14 and day 19 of AHC-6 group and day 19 mock where *n* = 5). Box plot centre lines represent medians, box limits represent upper and lower quartiles, and whiskers span minimum to maximum values, all points shown. Statistical significance between groups was evaluated by Kruskal–Wallis (pairwise) analysis. *P < 0.05, after Benjamini–Hochberg FDR correction. NS, not statistically significant. **c**, 2D PCoA plot of unweighted-UniFrac distance matrices before antibiotic treatment and at day 6 and day 19 post-infection. Pairwise PERMANOVA with Benjamini–Hochberg FDR correction were applied (*n* = 6 mice per group except day 19 mock where *n* = 5). Significance was detected for AHC-6 and Δ*npsB* (*q* = 0.002 (day 6) and *q* = 0.006 (day 19)), AHC-6 and mock (*q* = 0.006 (day 19)) and Δ*npsB* and mock (*q* = 0.006 (day 19)). Ellipses represent the 95% confidence interval. **d**, Box plots (centre lines represent medians, box limits represent upper and lower quartiles, and whiskers span minimum to maximum values, all points shown) represent pairwise comparison of unweighted-UniFrac distances to baseline (pre-antibiotic) for each group over time (*n* = 6 mice per group except day 14 and day 19 of AHC-6 group and day 19 mock where *n* = 5). **e**, Relative abundance (%) of bacterial genera significantly amplified or suppressed by toxigenic AHC-6 on day 19 (LEfSe analysis, linear discriminant analysis (LDA) >2) plotted for all days as bubbles; genera of the phylum Bacteroidota are indicated (§). **f**, Alpha diversity (observed richness) calculated for Bacteroidota. Box plots (centre lines represent medians, box limits represent upper and lower quartiles, and whiskers span minimum to maximum values, all points shown) compare observed sequence variants over time (*n* = 6 mice per group except day 6 and day 14 of AHC-6 group where *n* = 5). Statistical significance was evaluated by Kruskal–Wallis (pairwise) comparisons. *q < 0.05, after Benjamini–Hochberg FDR correction. Source data

**Extended Data Fig. 3:**
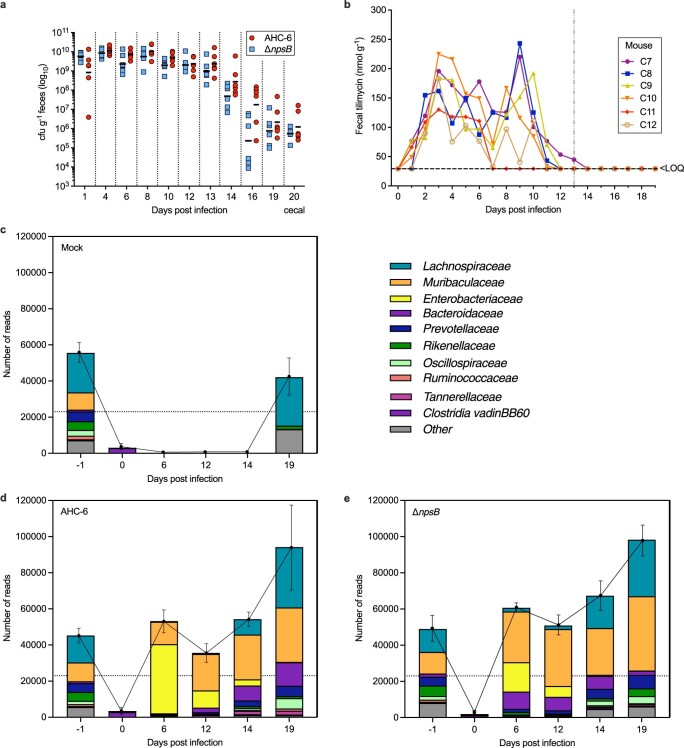
Total bacterial reads obtained over time for uninfected mock control and *K. oxytoca* harboring communities. **(a)** Fecal densities of *K. oxytoca* over time in colonized mice (n = 6 mice/group). Shown are values for individual animals and the geometric mean (bars) of cfu g^−1^ feces. No *K. oxytoca* or TM was detected in mock controls (n = 6). **(b)** Daily fecal TM concentrations per *K. oxytoca* AHC-6-infected mouse (n = 6). Dotted line indicates withdrawal of Curam 13 days post infection. LOQ denotes limit of quantification for TM. **(c-e)** Number of reads obtained for the 10 most prevalent bacterial families detected in feces of **(c)** uninfected mice (mock control, n = 6), **(d)** mice infected with AHC-6 (n = 6) or **(e)** Δ*npsB* (n = 6). Reads belonging to other bacterial families were combined (Other). Bars represent number of reads per bacterial family. Line plots represent mean total bacterial reads (±SEM). Datasets were analyzed at equal sequencing depth of 23,000 reads (dotted line). Source data

**Extended Data Fig. 4:**
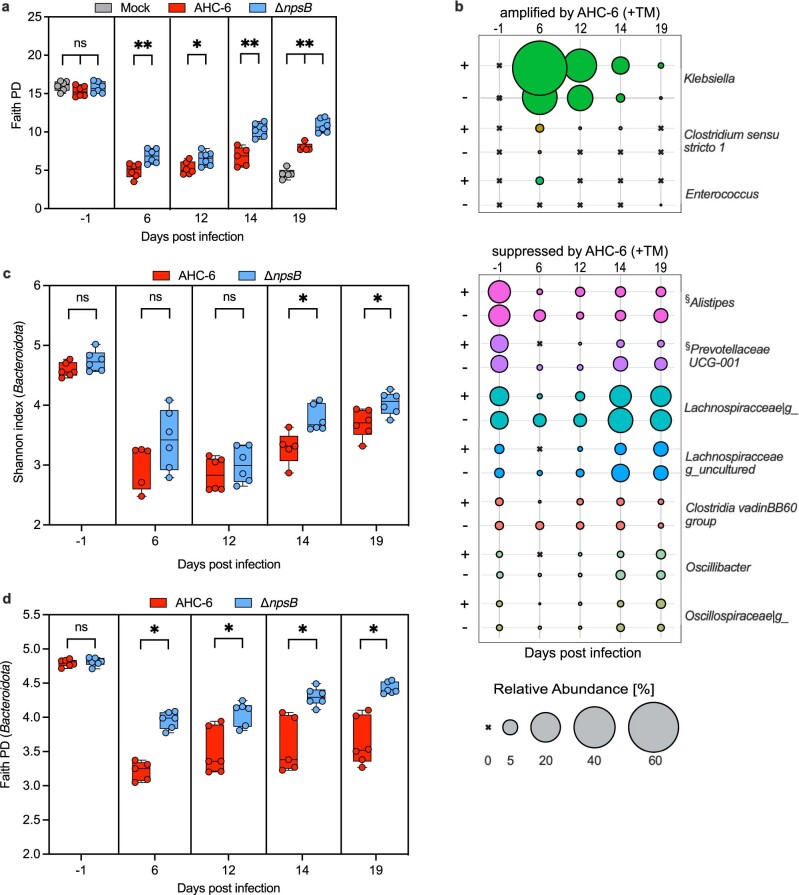
Genotoxic *K. oxytoca* reduce abundance and diversity of Bacteroidota taxa. **(a)** Boxplots compare median phylogenetic diversity values (Faith PD) for each group before infection and over time post-infection (n = 6 mice/group except day 14 and day 19 for AHC-6 and mock on day 19 where n = 5). Statistical significance was evaluated by Kruskal-Wallis (pairwise) analysis. *q < 0.05, **q < 0.01 after Benjamini-Hochberg FDR correction, (ns) not statistically significant **(b)** Relative abundance (%) of bacterial genera significantly amplified or suppressed by toxigenic AHC-6 on day 6 (LEfSe analysis, LDA > 2) plotted for all days as bubbles; genera of the phylum Bacteroidota are indicated (§). Alpha-diversity indices (**c**; Shannon) and (**d**; Faith PD) calculated for Bacteroidota over time shown as boxplots (n = 6 mice/group except day 6 and day 14 for AHC-6 where n = 5). Statistical significance was evaluated by Kruskal-Wallis (pairwise) analysis. *q < 0.05, after Benjamini-Hochberg FDR correction, (ns) not statistically significant. **a,c,d**, Boxplot center lines represent medians, box limits represent upper and lower quartiles, whiskers span min. to max. values, all points shown. Source data

We analysed the longitudinal effects of TM on community structure in detail. Mice were stably colonized with *K. oxytoca* until day 13 when Curam was withdrawn (Fig. 3a and Extended Data Fig. 3a). High faecal concentrations of TM were present from days 3 to 10 (Extended Data Fig. 3b). The impact of Curam on bacterial diversity is illustrated in Fig. 3b. Before treatment, each cohort showed the same high taxonomic richness (Fig. 3b), but reliable datasets were not retrieved for any group on day 0 (24 h post-Curam) (Extended Data Fig. 3c–e). Nonetheless, comparison of the total reads retrieved over time showed no lasting reduction or differences between the infection groups (Extended Data Fig. 3d,e). Notably, *K. oxytoca* supported comparatively richer communities than the mock control throughout the experiment independently of TM production (Fig. 3b). That finding is consistent with other in vivo studies showing that ß-lactamase-producing bacteria, such as *K. oxytoca*, lower the effective concentration of amoxicillin in the gut and thereby facilitate the growth of susceptible bacteria^34,35^. Importantly for this analysis, the additional presence of abundant TM at day 6 limited the number of observable features significantly compared with non-producers. This difference was eliminated by day 12 when faecal TM was no longer measured. Richness continued to increase for both colonized groups after day 12, yet the community succession occurring in this timescale remained significantly less diverse following TM exposure (Fig. 3b). Faith’s phylogenetic diversity measured over this timeframe revealed that TM not only lowered diversity but also significantly shifted the composition of the succession communities (Extended Data Fig. 4a). To reveal phylogenetic differences between these communities, we calculated unweighted-UniFrac distances (Fig. 3c). The PCoA shows that the initial communities in all cohorts before Curam treatment were highly similar. After day 6 of *K. oxytoca* colonization with and without TM exposure, the composition of the AHC-6- and Δ*npsB*-containing communities diverged strongly. By day 19, the composition of the successive bacteria in each cohort recovered some features of the pre-Curam state (Fig. 3d), but remained significantly distinct from each other (Fig. 3c). Recovery towards pre-treatment beta diversity in this timescale was most pronounced in animals carrying the non-genotoxic strain (Fig. 3d).

Differentially abundant bacterial genera in the toxicogenic versus mutant cohorts over time belong to the Bacteroidota and Bacillota phyla (Fig. 3e and Extended Data Fig. 4b). *Bacteroides* was particularly depleted when TM concentrations were highest, consistent with the pronounced suppression of the Bacteroidota in human samples (Fig. 1a,b). Genera amplified by TM included *Enterococcus* and *Clostridium sensu stricto* *1*. TM-dependent differences in the post-antibiotic succession also involved mainly members of the Bacteroidota. *Bacteroides* and Tannerellaceae_g populations expanded to higher relative abundance following TM exposure, while *Prevotellaceae UCG-001* and *Odoribacter* prevailed in the absence of TM (Fig. 3e). Given the observed volatility within the Bacteroidota, we analysed the diversity indexes for this phylum specifically. The initial composition in both cohorts was comparable. yet TM exposure resulted in significantly less richness, reduced evenness and strongly influenced the taxonomic structure of the Bacteroidota present in the succession communities compared with the TM-free cohort over this timescale (Fig. 3f and Extended Data Fig. 4c,d).

Taken together, we demonstrated that faecal microbial communities of specific-pathogen-free mice diverged strongly and significantly after a single event of TM exposure. The resulting composition and altered inter-species interactions in turn shifted the trajectory of ecological succession post-exposure. It is intuitive that the impact of TM on the gut ecosystem is contingent upon the initial community structure. Given the complexity and extreme intra- and inter-individual variation in the human gut microbiome, divergent outcomes can be expected for each episode of gut intoxication^36,37^.

### TM is a mutagen and antagonizes residents of the same niche

**Fig. 4:**
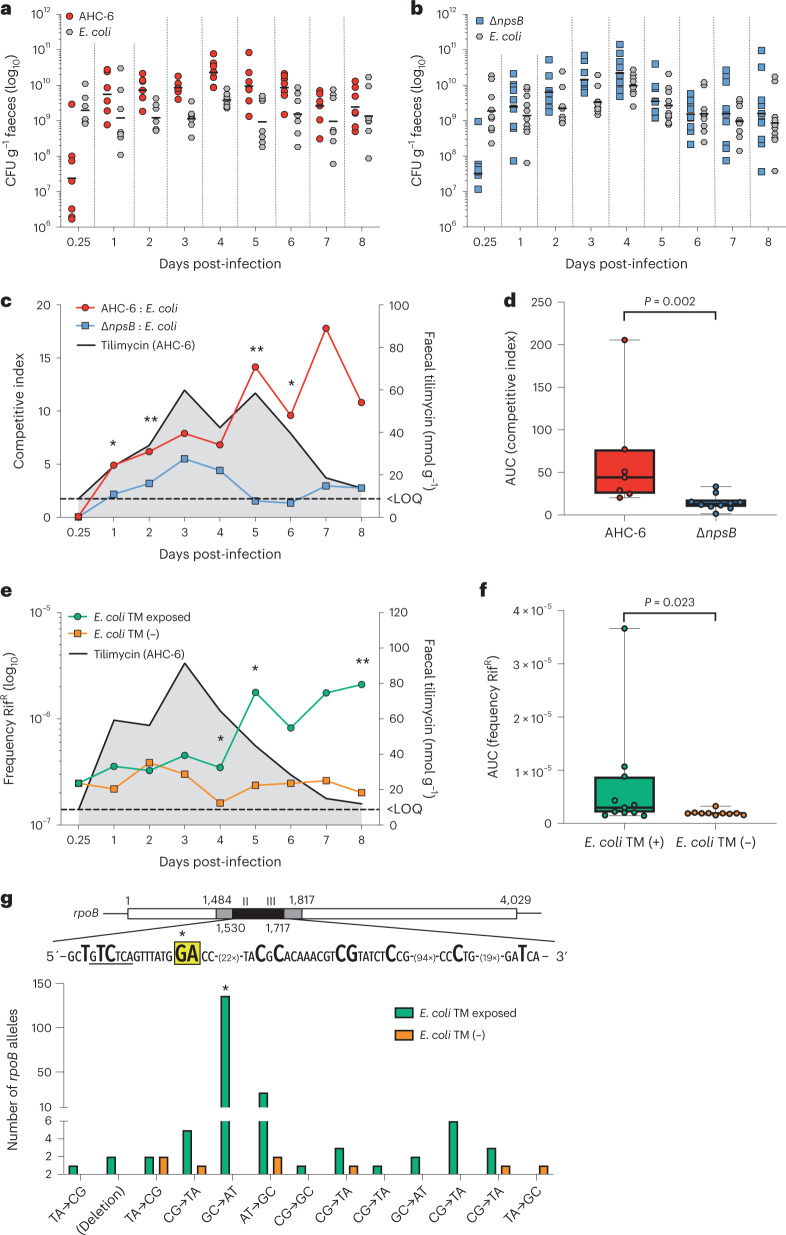
Genotoxic *K. oxytoca* antagonize competitors and drive mutational resistance emergence. **a**,**b**, Faecal levels of competing bacteria *K. oxytoca* AHC-6 and *E. coli* B13Sm (*n* = 7) (**a**) or *K. oxytoca* Δ*npsB* and *E. coli* B13Sm (n = 10) (**b**) were monitored daily. Shown are individual values and the geometric mean (bars) of CFU g^−1^ faeces. **c**, Comparison of calculated competitive indices of *K. oxytoca* versus *E. coli* in the presence (AHC-6, *n* = 7) or absence (Δ*npsB*, *n* = 10) of TM. Plotted are mean values based on CFU g^−1^ faeces. Black line with grey shading marks TM concentrations (mean) over time for AHC-6. **d**, Box plots (centre lines represent medians, box limits represent upper and lower quartiles, and whiskers span minimum to maximum values, all points shown) of calculated area under the curve (AUC) of competitive indices for each group (AHC-6, *n* = 7 and Δ*npsB*, *n* = 10). Kruskal–Wallis (pairwise) analysis (*P* = 0.002). **e**, Frequencies of Rif^R^ emergence in *E. coli* in TM-exposed (*n* = 11) or TM-free (*n* = 10) mice. Shown is the mean mutation frequency over time post-infection and TM concentration over time for AHC-6 (black line with grey shading). In **c** and **e**, LOQ indicates limit of quantification for TM. Statistical significance (two-sided) was evaluated by Mann–Whitney test (*P < 0.05, **P < 0.01). **f**, Box plots (centre lines represent medians, box limits represent upper and lower quartiles, and whiskers span minimum to maximum values, all points shown) of calculated AUC of mutation frequencies for groups ± TM exposure (+TM, *n* = 11; −TM, *n* = 10). Kruskal–Wallis (pairwise) analysis (*P* = 0.023). **g**, Mutation spectrum in *rpoB* of resistant *E. coli* (*n* = 189 versus *n* = 8 alleles) isolated from TM-exposed and TM-free mice, respectively. Gene map identifies region sequenced. Frequencies of each site-specific mutation (bold) are shown. (*) Guanine at position 1,546 highlighted in yellow identifies predominant post-exposure mutation. Source data

TM’s impact on community composition in the gut may reflect direct suppression of different bacterial populations, consistent with the broad activity range we observed in vitro. Other changes induced by TM may progress through the network of ecological interactions, which will involve species not directly affected. To explore the underlying mechanisms we first introduced a single specific competitor for co-colonization with *K. oxytoca* in this model. For this purpose, we isolated a strain of *E. coli* (B13) from the caecal content of a mouse from the antibiotic control group in the preceding experiment (Fig. 2). This strain was well suited for the study as it is mouse adapted, resistant to Curam and exerts no apparent pathology. Mice were inoculated 1 day post-Curam with a 1:1 suspension of a streptomycin-resistant mutant of B13 (B13Sm) and either AHC-6 (*n* = 7) or Δ*npsB* (*n* = 10). The dynamics of colonization and TM production were monitored over time (Fig. 4a–c).

Expansion of *E. coli* outpaced *K. oxytoca* initially (6 h post-infection), but the relationship was reversed after 24 h. The *K. oxytoca* strains outcompeted *E. coli* B13Sm generally. However, toxin producers reached significantly higher competitive indices in the dual infection than non-producers (Fig. 4c,d). This finding agrees well with the previous experiments where higher relative abundances of genotoxic *K. oxytoca* (wild type and complementation group) than non-producers were attained in mice following the peak of TM production (Extended Data Figs. 2e and 4b). We conclude that TM confers producing strains an ecological advantage.

To distinguish whether the negative impact on *E. coli* observed in vivo resulted from a direct interaction with TM, we focused next on TM’s genotoxic activity. TM is a DNA-alkylating agent with a preference for *N*2 of guanine^25^; thus, genome-wide lesions can be expected, resulting in some cases in reduced fitness or increased mortality. Mammalian cells exposed to TM activate a DNA damage response, accumulate lesions and undergo genome fragmentation and apoptosis^20^. Bacteria react to DNA damage with the conserved SOS stress response. The repair processes involve error-prone DNA polymerases, which typically cause elevated mutation rates^38,39^. We reasoned that, if the TM-mediated inhibition of *E. coli* was direct, we should detect a TM-dependent increase in mutations occurring in vivo. Mutation is a major mechanism driving antibiotic resistance emergence in bacteria^40^. We therefore monitored de novo emergence of rifampicin resistance (Rif^R^) in *E. coli* B13Sm during gut passage. Resistance emerged more frequently in animals colonized with genotoxin producers (*n* = 11) compared with non-producers (*n* = 10) (Fig. 4e,f). In this in vivo model we would not expect Rif^R^ *E. coli* to undergo positive selection. The numbers of mutants isolated from the Δ*npsB* infection group remained stable over time, supporting our premise. The significant rise in Rif^R^ over time detected with AHC-6 therefore probably reflects the continuous emergence of new resistance. The mutation spectrum observed in *rpoB* genes of resistant faecal isolates was diverse with or without TM production (Fig. 4g and Supplementary Table 1). The overlap in these single base-pair substitutions may reflect the background of ‘pre-exposure’ mutations occurring in vivo during Curam treatment. The high number of G:C to A:T transitions occurring at position 1,546 during TM exposure in vivo (136/189 alleles) identifies the predominant ‘post-exposure’ signature. This substitution in sequence context TGGAC agrees well with the preferred reactivity of TM with guanines flanked by purines^25^. The absence of this signature in resistant alleles generated without luminal TM adds evidence that resistance emergence observed with murine hosts indeed reflects intestinal events and not the in vitro selection (Supplementary Table 1).

### DNA damage drives TM-dependent resistance emergence

**Fig. 5:**
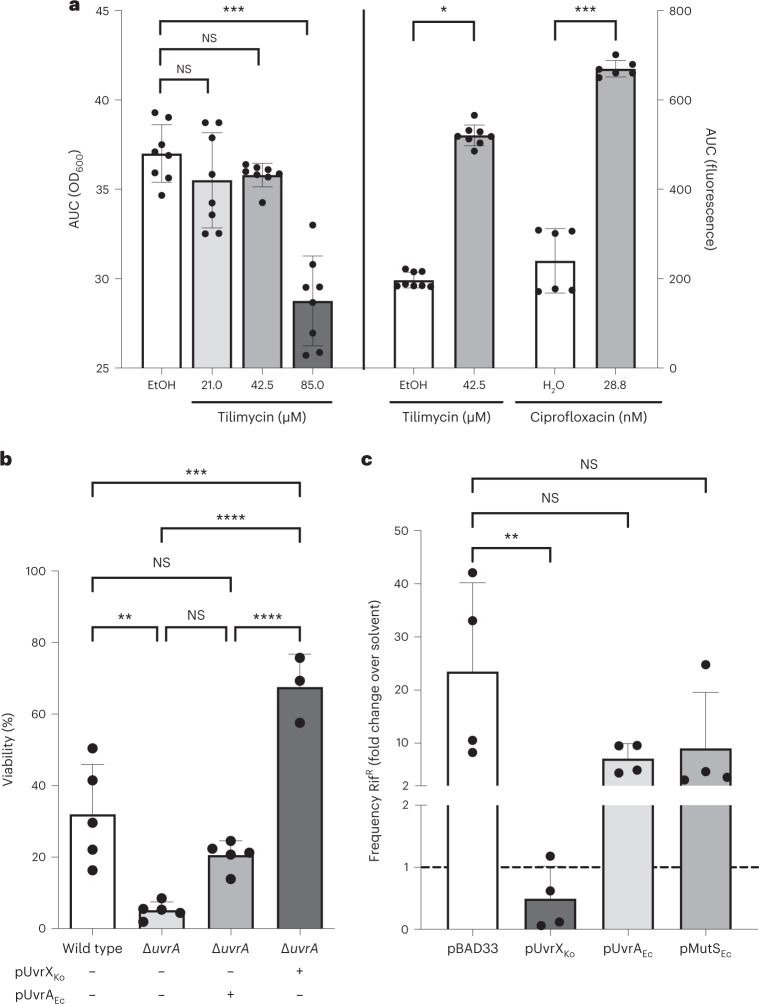
*K. oxytoca* immunity gene *uvrX* alleviates DNA stress ectopically. **a**, Calculated AUC values for growth (left) and fluorescence intensity (right) of *E. coli* MG1655 [pUA66-P_*recA*_-gfp] cultured for 15 h in CASO medium supplemented with TM or ciprofloxacin (Extended Data Fig. 5a,b). P_*recA*_-gfp induction for subinhibitory concentrations of TM and ciprofloxacin are shown (mean (±SD) and individual values of independent measurements, *n* = 8 (EtOH and TM); *n* = 6 (H_2_O and ciprofloxacin)). Statistical difference between genotoxin and EtOH control was evaluated by Kruskal–Wallis with Dunn’s test; *P < 0.05, ***P < 0.001; NS, not statistically significant. **b**, Survival of *E. coli* after TM exposure (85 µM; 2 h) relative to EtOH treatment. Shown are mean values (±SD) for parent strain *E. coli* JW0001_1 and JW4019_2 (Δ*uvrA*) with empty vector (*n* = 5 independent experiments each), and JW4019_2 (Δ*uvrA*) carrying pUvrX_Ko_ (*n* = 5) or pUvrA_Ec_ (*n* = 3). **c**, *E. coli* MG1655 was grown in the presence of 42.5 µM TM for 16 h. Change in frequency of resistance emergence (Rif^R^) in cultures carrying indicated expression plasmids. Shown are mean values (±SD) of the relative increase in resistance over solvent control (*n* = 4 all groups). Mutation frequency of solvent control was set to 1 (dashed line). In **b** and **c**, one-way analysis of variance evaluated statistical significance with Bonferroni correction. **P < 0.01, ***P < 0.001, ****P < 0.0001. Source data

**Extended Data Fig. 5:**
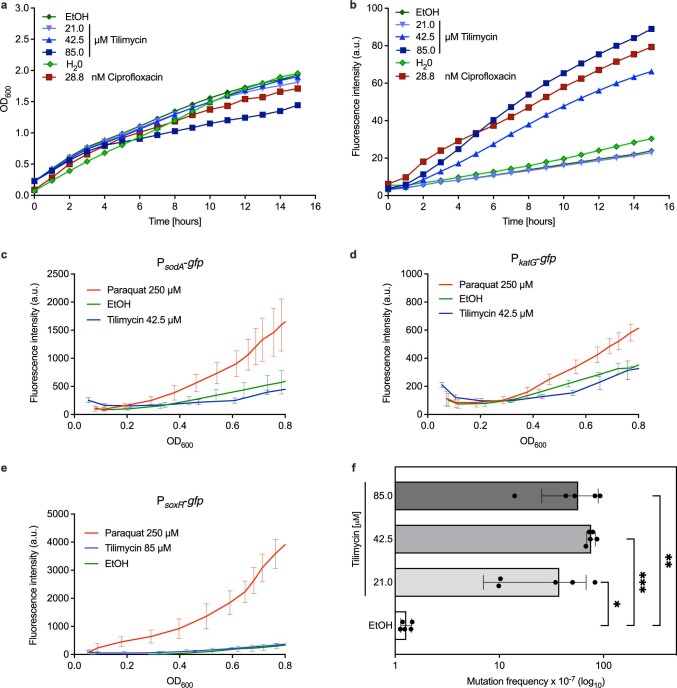
TM induces the *E. coli recA* promoter and resistance emergence in vitro but not redox sensitive genes. Mean **(a)** OD_600_ and **(b)** fluorescence were measured during growth of *E. coli* MG1655 [pUA66-P_*recA*_-gfp] in CASO medium supplemented with different concentrations of TM, ciprofloxacin as positive control, and the respective solvent in independent measurements [n = 8 (EtOH and TM); n = 6 (H_2_0 and ciprofloxacin)]. **(c, d, e)** OD_600_ and fluorescence were measured over time for *E. coli* MG1655 transformed with **(c)** pUA66-P_*sodA*_-gfp **(d)** pUA66-P_*katG*_-gfp and **(e)** pUA66-P_*soxR*_-gfp grown in presence of TM (42.5 µM or 85 µM), positive control paraquat or EtOH. The mean (±SD) of 3 independent measurements is shown. **(f)** Frequency of Rif^R^ emergence in *E. coli* MG1655 after 16 h TM exposure. Shown is the mean (±SD) mutation frequency (Rif^R^ over total cfu) and individual values of 5 independent experiments. Ordinary one-way ANOVA with Holm-Šídák correction evaluated statistical significance. (*p < 0.0202, **p < 0.0022, ***p < 0.0002). Source data

Although TM-mediated genotoxicity very likely underlies the observed mutational emergence, it is also known that some bacteria respond to low levels of antibiotics with induction of SOS and the general stress response, reactive oxygen stress and a hypermutator phenotype irrespective of the drugs’ molecular targets^29,41–44^. To characterize the *E. coli* response to TM in detail, we recapitulated the mutational processes in vitro. *E. coli* K12 strain MG1655 or this strain carrying a fused *recA* promoter–green fluorescent protein (P_*recA*_*-gfp*) reporter plasmid was cultured aerobically in concentrations of TM corresponding to peak in vivo levels or below, or with a ciprofloxacin control (Fig. 5a and Extended Data Fig. 5a,b). Cirz et al.^39^ showed that repair of ciprofloxacin-mediated DNA damage results in derepression of SOS-regulated polymerases, which in turn generate resistance-conferring mutations^39^. Significant induction of P_*recA*_ was observed with subinhibitory levels of TM (42 µM) and in ciprofloxacin controls. To assess whether subinhibitory TM treatment could induce target-independent DNA damage in *E. coli*, we assayed for increased intracellular oxidative stress using reporter fusions of the promoters of *sodA*, *katG* and *soxR*. Although the paraquat control was able to stimulate these redox-sensitive promoters, TM did not (Extended Data Fig. 5c–e). Significantly more Rif^R^ emerged in *E. coli* treated with all concentrations of TM tested compared with controls (Extended Data Fig. 5f).

Genotoxin-producing organisms can be expected to protect themselves from autotoxicity^45^. We tested the hypothesis that a self-resistance determinant (*uvrX*) co-localized on the *til* genomic island in *K. oxytoca* should effectively protect *E. coli* from TM-mediated DNA damage. Structural modelling of UvrX encoded directly upstream of the *til* biosynthesis genes predicted a folded protein most similar to UvrA2 from *Deinococcus radiodurans* (PDB ID: 2VF7). Deletion of the *E. coli* homologue *uvrA* induced hypersensitivity to TM (Fig. 5b). Ectopic expression of the putative resistance gene *uvrX* in the Δ*uvrA* mutant indeed increased survival to TM significantly. Moreover, the frequency of TM-dependent Rif^R^ emergence in vitro was reduced 25-fold by *uvrX* (Fig. 5c). Notably, however, it was not significantly altered by overexpression of the *E. coli* homologue *uvrA*. The mutator phenotype driven by general antibiotic-induced stress arises in part owing to RpoS-dependent downregulation of mismatch DNA repair efficiency^42^. Although restoring MutS levels has been shown to lower mutation rates caused by several antibiotics, no significant change in TM-dependent resistance emergence occurred by overproducing MutS in *E. coli* (Fig. 5c). These findings indicate that the self-resistance determinant UvrX is better adapted to alleviate TM-mediated genotoxicity than general DNA repair pathways.

The sum of these findings supports the conclusion that TM generates mutational resistance mainly via direct DNA damage rather than target-independent pathways.

### Mutational evolution of pathogenic strains

**Extended Data Fig. 6:**
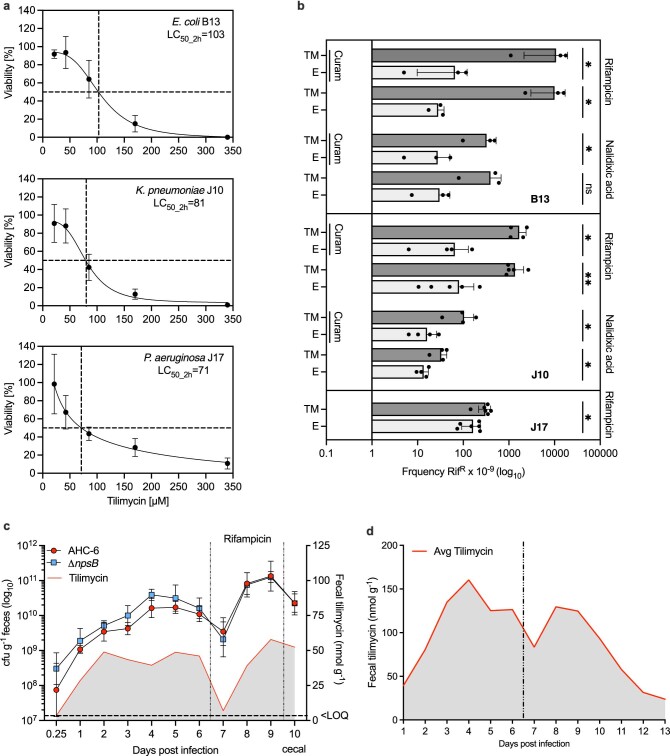
TM expands the resistance repertoire of pathogens belonging to *Pseudomonadota*. **(a)** Lethal concentration (50% mortality; LC_50_2h_) of TM for *E. coli* B13, *K. pneumoniae* J10, and *P. aeruginosa* J17 after 2 h exposure. Data points represent the mean (±SD) of 5 replicates. **(b)** *E. coli* B13, *K. pneumoniae* J10, and *P. aeruginosa* J17 were exposed to TM and EtOH (E) with or without Curam for 2 h. Treatments: *E. coli* (85 µM TM, 30 µg ml^−1^ Curam), *K. pneumoniae* (85 µM TM, 60 µg ml^−1^ Curam), and *P. aeruginosa* (21.5 µM TM). Frequencies of resistance acquisition for rifampicin and nalidixic acid are shown as mean (±SD) observed in each group (n = 3 for B13, n = 4 or 5 for J10, n = 6 for J17). Statistical differences between TM and EtOH treatments were evaluated with two-sided Mann-Whitney test, *p < 0.05, **p < 0.01, (ns) not statistically significant. **(c)** Fecal and cecal densities of *K. oxytoca* in dual infection with *K. pneumoniae* (n = 4 mice/group). Shown are geometric means (±SD) of cfu g^−1^ feces/cecal content (day 10) and average concentrations (mean) of TM for the AHC-6 group over time (red line with grey shading). No TM was detected in the Δ*npsB* group. **(c and d) Renewed surge in TM secretion triggered by change in course or effective dose of antibiotics. (c)** Dotted line indicates replacement of Curam 6 days post-infection with rifampicin selection. LOQ denotes limit of quantification for TM. **(d)** TM secretion stimulated by a fresh Curam solution on day 6 **(**dotted line). Shown is shift in fecal TM values (red line with grey shading) after antibiotic renewal. Average values from experiments Figs. 2 and 3 (n = 10 mice). Source data

Our findings show that TM producers have the capacity to antagonize competitors with mutational burden. Direct outcomes of those interactions can include the rise of genetic variants and spread of mutant alleles in commensal and pathogenic strains alike. As gastrointestinal populations are sources for infections^46^, the clinical implications are manifold. To underline this point empirically, we next asked whether the TM-dependent evolution of resistance observed with *E. coli* in vivo and in vitro could also be detected in opportunistic pathogens, specifically members of the ESKAPE group, which is responsible for most nosocomial infections^47–49^. Mutational evolution of these pathogens during colonization has implications for tissue invasion, systemic dissemination and antibiotic resistance emergence. Faecal isolates of *Pseudomonas aeruginosa* J17 and *Klebsiella pneumoniae* J10 originating from the human donors sampled in (Fig. 1) and murine isolate B13 were treated with TM in vitro to determine the concentrations causing 50% lethality in 2 h (LC_50_) (Extended Data Fig. 6a) and minimal inhibitory concentrations (MIC). Concentrations at or below the LC_50_ were used alone and in combination with Curam to promote genetic variation (Extended Data Fig. 6b). Resistance to rifampicin and nalidixic acid in *E. coli* B13 and in *K*. *pneumoniae* J10 emerged as a result (Extended Data Fig. 6b). Exposure of *P. aeruginosa* isolate J17 to TM also added another resistance determinant to a human isolate already endowed with multiple antibiotic resistances (Extended Data Fig. 6b and Supplementary Table 2). These findings verify that opportunistic pathogens exposed to TM sustain mutations.

**Fig. 6:**
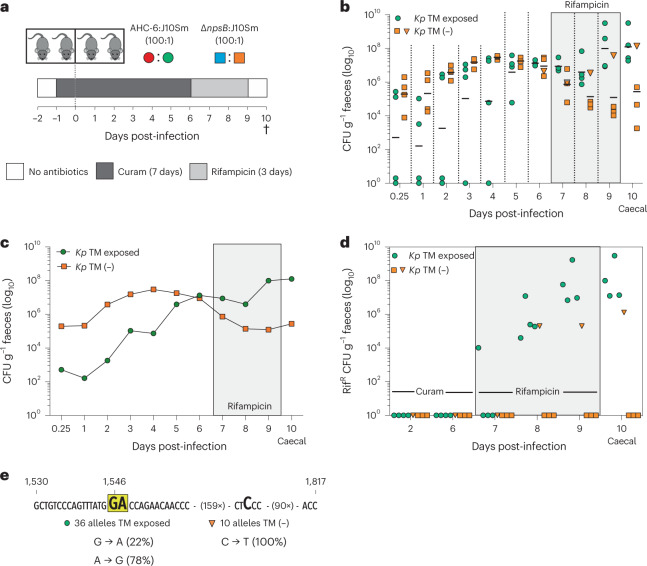
Cytotoxic *K. oxytoca* drives resistance emergence of *K. pneumoniae* in vivo. **a**, Dual-antibiotic treatment study design. Cohorts received Curam 24 h before co-gavage with *K. pneumoniae* J10SM and toxigenic *K. oxytoca* AHC-6 or TM-/TV-deficient Δ*npsB*. Curam was replaced by rifampicin 6 days post-infection. Mice were killed on day 10 after 3 day rifampicin treatment and 24 h post-antibiotic recovery. **b**, Faecal (days 0.25 to 9) and caecal (day 10) levels of *K. pneumoniae* J10Sm (*Kp*) competing with *K. oxytoca* AHC-6 (*n* = 4) or Δ*npsB* (*n* = 4). Shown are individual values and the geometric mean (bars) of CFU g^−1^ faeces. Orange triangle depicts CFU for single mouse of the Δ*npsB* group excreting Rif^R^ J10Sm. **c**, Geometric means of data shown in **b**. **d**, Faecal (days 0.25 to 9) and caecal (day 10) numbers of Rif^R^ *K. pneumoniae* for the two groups. Shown is CFU g^−1^ for each mouse; orange triangles, same sample as in **b**. **e**, Mutation spectrum in *rpoB* of Rif^R^ *K. pneumoniae* (*n* = 36 versus *n* = 10 alleles) isolated from TM-exposed and TM-free mice, respectively. Guanine at position 1,546 and adenine 1,547 (yellow highlight) were the predominant post-exposure substitutions. Source data

Expansion of the reservoir of resistance determinants in vivo may cause colonized patients to shed multi-resistant opportunistic pathogens in health care settings. Outbreaks of resistant pathogens may also result from their direct selection through antibiotic interventions. To model these scenarios, we performed a dual infection study (Fig. 6a) in the mouse combining *K*. *pneumoniae* J10 with toxigenic (*n* = 4) or mutant Δ*npsB K. oxytoca* (*n* = 4). Like *K. oxytoca*, *K. pneumoniae* is intrinsically resistant to ß-lactams. It persists in the host under the same dysbiotic conditions and competes with *K. oxytoca* for niche occupation^50^. We sought to keep the number of colonizing *K*. *pneumoniae* low by administering mice 100-fold fewer J10 than *K. oxytoca*. When competing with toxin-deficient Δ*npsB* bacteria, *K*. *pneumoniae* colonized all mice within 24 h. By contrast, when TM was produced in the intestine, establishment of *K*. *pneumoniae* was delayed for 3–5 days in some animals (*n* = 2) (Fig. 6b). Rif^R^ was not detected in faecal *K*. *pneumoniae* of either cohort for 6 days (Fig. 6c). To capture rare *K*. *pneumoniae* mutants that may have emerged, we changed the antibiotic intervention from Curam to rifampicin. All mice (*n* = 4) in the toxigenic cohort excreted Rif^R^ *K*. *pneumoniae* within 24–48 h. In contrast only one mouse of the toxin-free cohort shed Rif^R^ *K*. *pneumoniae* after 72 h of direct selection. The mutation spectrum of resistance-conferring *rpoB* alleles associated with each cohort is shown in Fig. 6d. The identical signature mutation for TM observed previously in *E. coli* (Fig. 4g and Supplementary Table 1) was also observed in *K*. *pneumoniae* after in vivo exposure. Those alleles emerging from the gut in the absence of TM carried a different single nucleotide substitution in a distinct sequence context (Fig. 6d).

We conclude that outgrowth of genotoxic *Klebsiella* during antibiotic therapy increased gene mutation frequency in the gut and promoted mutational evolution of colonizing opportunistic pathogens and emergence of de novo resistance. In sum, these findings show that genotoxin TM influences the evolution of the gut microbiota not just by imposing selection pressures upon it, but by altering the mechanisms of evolution.

## Discussion

TM is active in both human and bacterial cells; thus, off-target effects of antibiotic use put patients at risk for species depletion, antibiotic-associated diarrhoea and intestinal disease. Genotoxic *K. oxytoca* and *K. grimontii* are early colonizers of the human intestine. Microbiome development monitored during the first month of life for a large cohort of UK infants found that carriage rates for *K. oxytoca* alone reached 9% in neonates delivered vaginally and 20% following caesarian section^15^. Genotoxic species may constitute a larger fraction of the gut microbiome in infants than in adults, but cytotoxicity is common to isolates of healthy adult (42%) and infant carriers (49%) (refs. ^51,52^). The incidence of colitis caused by bacterial overgrowth in neonates—resulting in some cases of necrotizing enterocolitis^24^—as well as AAHC in children, adults and the elderly^23^ underscore the point that humans are long-lived hosts and carriers may experience multiple episodes of toxicity over a lifetime.

The current study demonstrates that genotoxic *Klebsiella* spp. impact microbial evolution in the mouse and potentially in human hosts. This is possible both during a transient bloom—as recapitulated in this antibiotic dysbiosis model—or through lower exposure over a longer timescale when producer densities return to homeostatic levels. Our findings may be particularly relevant for neonates. The microbial community in the newborn gut is dynamic during the first years of life^53–57^. Maturation of these early communities contributes importantly to a child’s metabolic and immune development^36,58,59^, whereas disruptions predispose children to immune-mediated disorders^60,61^ and expand the reservoir of antibiotic resistance genes they carry^16^. Our findings thus raise concern that early life experience with antibiotics, medications or dietary changes that trigger dysbiotic outgrowth or otherwise affect ecological opportunity will add the additional pressure of TM exposure in the infant gut. In such cases, increased microbial volatility and concomitant loss of community resilience could further impair this critical phase of microbial maturation.

Incidents of TM-induced destabilization and mutational evolution of the gut ecosystem may be repetitive events over a lifetime. Health care settings and factors driving dysbiotic overgrowth of *K. oxytoca*—from chemotherapies to immunodeficiencies—pose challenges for colonized patients at all life stages^62^. A dysbiosis-induced event of TM secretion is self-limiting in patients with AAHC^20^ and as shown here in the mouse (Figs. 2b and 3a), yet when we applied a new insult, such as a higher dose or a different class of antibiotic, strong TM production was again induced in colonized mice (Extended Data Fig. 6c,d). The mutagenic environment created by a single asymptomatic event of genotoxic *Klebsiella* outgrowth was sufficient to expand the array of antibiotic resistance determinants available to gut bacteria. Colonization with genotoxic *Klebsiella* spp. may thus put individuals at risk for future resistant infections originating from the commensals and opportunistic pathogens they carry.

In addition to driving resistance emergence in vivo, hypermutator activity provoked under these conditions would be expected to have broad evolutionary impact on gut bacteria. The general nature of TM’s reactivity towards guanine coupled with the susceptibility of numerous and diverse taxa underscores the potential for widespread genetic variation within the host microbiota. Single mutations or combinations thereof that accumulate during the timescale of exposure may accelerate adaptations to changes in environmental and community contexts or upon introduction of novel abiotic stressors. The outcome of structural and functional variation will vary between individuals and within individuals over time, shaped by the size of the *til*^+^ population and selective forces in changing gut environments.

Repeated antibiotic perturbation is known to challenge the long-term stability of the human distal gut microbiota^37^. This study reveals the concomitant risk of elevated mutation and destabilization of the gut ecosystem that results from transient exposure to a broad-spectrum genotoxin. The consequences of these combined forces on the resilience of the gut community remain unknown. Our findings provide a good starting point to ask whether and how the bacterial production of drug-like molecules in humans can be regulated to reduce intestinal damage and increase both community resistance and resilience.

## Methods

### Ethics statement

Nine healthy human volunteers were recruited at the Medical University of Graz. Written informed consent was obtained from all participants, and stool samples were collected and pseudonymized by Dr C. Högenauer. The study was conducted according to federal guidelines, the local ethics committee regulations and the Declaration of Helsinki. This study was approved by the institutional review board of the Medical University of Graz (17–199 ex 05/06).

All mouse studies were performed in accordance with the Commission for Animal Experiments of the Austrian Ministry of Science (GZ BMWFW-66.007/0002-WF/V/3b/2017 and BMWFW-39/12175ex2017/18) and the local ethics committee of the University of Graz. Mice were housed in specific-pathogen-free conditions in individually ventilated cages and maintained on a 12 h light/dark cycle at 21 °C and 48% humidity. Food and water were offered ad libitum. Mice were monitored daily to record their weight and stress levels and killed at the study endpoint with isoflurane and cervical dislocation.

### Synthesis of *cis*/*trans*-TM

For the current study, the synthetic TV batch was identical to that in ref. ^12^, but TM was re-synthesized. Three equally sized batches were prepared consecutively as described in the following procedure: A dry 40 ml Schlenk tube with magnetic stirring bar was charged with a solution of (*S*)-(2-amino-3-hydroxyphenyl)(2-(hydroxymethyl)pyrrolidin-1-yl)-methanone (200 mg, 846 µmol, 1.0 eq.) in 2.8 ml of a 1:1 mixture (v/v) of anhydrous CH_2_Cl_2_/DMSO. The brown reaction mixture was cooled to 0 °C (ice bath), 370 µl (2.12 mmol, 2.5 eq.) DIPEA was added, and the orange solution was stirred for 5 min, after which 337 mg (2.12 mmol, 2.5 eq.) SO_3_·pyridine was added in one portion (brown solution). The reaction mixture was stirred at 0 °C for 60 min. After warming to room temperature, CH_2_Cl_2_ was removed under reduced pressure at 23 °C (Schlenk line with preceding cooling trap). The crude brown oil was divided into two high-performance liquid chromatography (HPLC) vials, and the product was isolated via preparative reversed-phase HPLC. The synthesis procedure was repeated two times. The pure fractions of all batches were pooled and lyophilized. The resulting yellow lyophilisate (197 mg) was further purified via flash column chromatography (SiO_2_, CH_2_Cl_2_/MeOH = 20:1 (v/v) to yield the pure title compound as a pale-yellow solid. Yield: 156 mg (666 µmol, 26%), pale-yellow solid C_12_H_14_N_2_O_3_ (234.26 g mol^−1^). R_*f*_ = 0.38 (CH_2_Cl_2_/MeOH = 10:1 (v/v); staining: CAM).

### Preparative and analytical HPLC method

RP Macherey-Nagel 125/21 Nucleodur 100-5 C18ec column (21 × 125 mm, 5.0 µm particle size, 100 Å pore size), column oven: 26 °C, flow rate 15 ml min^−1^, product detection: 220 nm; 0.0–7.0 min MeCN/H_2_O = 2:98 (v/v), 7.0–82.0 min linear increase to MeCN/H_2_O = 50:50 (v/v), 82.0–90.0 min linear increase to MeCN/H_2_O = 95:5 (v/v), 90.0–100.0 min hold MeCN/H_2_O = 95:5 (v/v), 100.0–102.0 min return to initial conditions. Analytical HPLC method: 40 °C, flow rate 0.7 ml min^−1^; 0.0–0.5 min MeCN/0.05% TFA = 10:90 (v/v), 0.5–9.0 min linear increase to MeCN/0.05% TFA = 100:0 (v/v), 9.0–11.5 min hold MeCN/0.05% TFA = 100:0 (v/v), 11.5–12.0 min return to initial conditions. HPLC purity (>95%) after prepHPLC and subsequent flash column chromatography is shown in Supplementary Information (Supplementary Table 3 and Supplementary Figs. 1 and 2).

### Growth inhibition of faecal bacteria

Fresh human stool (3 g) was suspended in 30 ml phosphate-buffered saline (PBS) and filtered through gauze. Flow-through plus a 10 ml PBS wash was collected and centrifuged at 550*g* for 15 min. The pellet was resuspended in 3 ml PBS. One 1 ml aliquot was incubated with 170 µM (40 μg ml^−1^) TM and a second 1 ml aliquot with solvent (EtOH, 0.5%) for 2 h at 37 °C under anaerobic conditions. Conversion of faecal quantities of analytes typical for mouse colonization (nmol TM g^−1^ or pmol TV g^−1^) to concentration was approximated by setting 1 g wet weight to 1 ml. Faecal samples (*n* = 16) of in-house-bred 8-week-old adult female C57Bl/6J mice housed in four cages (*n* = 4 mice each) were pooled for each cage, then homogenized in 16 ml PBS by vortexing with glass beads. Debris was allowed to sediment briefly before the supernatant was centrifuged at 550*g* for 15 min. The pellet was resuspended in 3.2 ml PBS, and 1 ml aliquots were incubated with 170 µM TM, 17 µM (6 μg ml^−1^) TV or EtOH for 2 h at 37 °C under anaerobic conditions. Treated human and mouse samples were washed, resuspended in 1 ml PBS. Serial dilutions were plated on selective and differential agar media (all purchased from Becton, Dickinson and Company, Austria) and incubated for ≤5 days to culture the following groups: bifidobacteria (*Bifidobacterium* agar (Bifido)); total anaerobic bacteria (Schaedler agar with vitamin K1 and 5% sheep blood (SCH)); anaerobic Gram-positive bacteria (Schaedler colistin–nalidixic acid agar with 5% sheep blood (CNA)); anaerobic Gram-negative bacteria (Schaedler kanamycin–vancomycin agar with 5% sheep blood (KV)); *B. fragilis* group (*Bacteroides* bile esculin agar (BBE)); aerobic Enterobacteriaceae (MacConkey agar without salt (MacC)). Viable cells for each sample and bacterial group were compared and expressed as percentage survival after toxin treatment relative to solvent. Selected colonies from all plates were subcultured and frozen at −80 °C. Isolates were identified via matrix-assisted laser desorption/ionization time-of-flight mass spectrometry analysis, VITEK MS (bioMérieux) and PCR amplification and sequencing of the 16S rRNA gene (V1–V4 region). Primers 27F (5′-AGAGTTTGATCCTGGCTCAG-3′) and 806R (5′-GGACTACCAGGGTATCTAAT-3′) were used with an annealing temperature of 57 °C. Sequences were analysed using Blastn (RefSeq_select).

### Bacterial strains and growth conditions

Bacterial strains used in this study are described in Supplementary Table 2. *K. oxytoca*, *K. pneumoniae* and *P. aeruginosa* were cultivated in tryptic soy broth (CASO) or on CASO agar and *E. coli* in LB broth or on LB agar. *K. oxytoca* strains used in mouse experiments were toxigenic *K. oxytoca gcvA*::Km (AHC-6), toxin-deficient *K. oxytoca npsB*::Tn5-*aphA* (Δ*npsB*), and *K. oxytoca npsB*::Tn5-*aphA* with an *npsB* expression vector (pNpsB)^33^. Ampicillin-resistant *E. coli* strain B13 was isolated from faeces of a mock-treated mouse. *K. pneumoniae* strain J10 and *P. aeruginosa* J17 were isolated from stool of one human donor. *E. coli* B13 and *K. pneumoniae* J10 were plated on CASO-Sm agar to generate the streptomycin-resistant derivatives B13Sm and J10Sm utilized in mouse experiments. Antibiotics (Sigma-Aldrich) were added to final concentrations of 100 μg ml^−1^ ampicillin (Amp), 40 μg ml^−1^ kanamycin (Km), 10 μg ml^−1^ chloramphenicol (Cm), 25 (*E. coli*) or 50 (*K. pneumoniae*) µg ml^−1^ streptomycin (Sm), and 100 (*E. coli*) or 200 (*K. pneumoniae* and *P. aeruginosa*) μg ml^−1^ rifampicin (Rif). All bacteria were grown at 37 °C.

### Monocolonization model and histology

Adult female C57BL/6NRj mice with SOPF status were purchased from Janvier Labs and infected with *K. oxytoca* at 8 weeks of age. In the first experiment, a total of 16 mice were used (Fig. 2 and Extended Data Fig. 2b–g). Sixteen mice were analysed histologically for Extended Data Fig. 2a. To analyse the longitudinal effects of TM on community structure in detail, we utilized 18 mice (Fig. 3 and Extended Data Figs. 3 and 4). Curam (1,000 mg amoxicillin/200 mg clavulanic acid, Sandoz) was administered in the drinking water (0.4 g l^−1^) 24 h before infection and until 13 days post-infection. Antibiotic solution was refreshed on day 6 post-infection. To prepare the inoculum, *K. oxytoca* AHC-6 and Δ*npsB* were grown for 24 h on CASO-Km agar and *K. oxytoca* AHC-6 + pNpsB on CASO-Km/Cm agar. A suspension of each strain was prepared by pooling single colonies in CASO broth to an optical density of 0.1 at 600 nm (OD_600_). Each mouse was gavaged with 10^7^ cells in 100 μl, or with 100 μl sterile CASO broth (mock). Viable bacteria in inocula were enumerated by plating on selection agar. Mice were kept on antibiotics for 2 weeks and killed after a 7 day antibiotic-recovery phase at day 21. Faecal pellets were collected daily for each mouse to analyse density of *K. oxytoca*, quantities of TM and TV, and microbiota composition. The absence of intrinsic *K. oxytoca* in all mouse cohorts was verified by stool culture before infection and randomly for some mice post-infection on Simmons citrate agar with 1% inositol. For histopathological evaluation, colon tissue was excised at sacrifice and epithelial damage was scored as described previously^22,63^.

### Dual colonization models

Curam was administered in the drinking water to in-house-bred 8-week-old adult female C57Bl/6J mice 24 h before infection. For the *E. coli*/*K. oxytoca* competition model, 21 mice were gavaged with a mixtures (10^7^:10^7^ cells in 100 µl CASO broth) of strains B13Sm and AHC-6 (*n* = 11) or B13Sm and Δ*npsB* (*n* = 10). To prepare the inocula, bacteria were grown on CASO agar for 24 h. Separate suspensions of each species were prepared in CASO broth to OD_600_ = 0.2 and mixed 1:1. Serial dilutions of gavage suspensions were plated on selective agar to determine CFU. Faecal densities of *K. oxytoca* were determined on CASO-Km plates, and *E. coli* B13Sm was quantified on CASO containing Sm and 40 μg ml^−1^ 5-bromo-4-chloro-3-indolyl-β-d-galactopyranoside. Rif^R^ *E. coli* were selected on CASO-Sm/Rif plates.

For dual colonization of mice with *K. oxytoca* and *K. pneumoniae*, eight in-house-bred 8-week-old adult female C57Bl/6J mice were housed in pairs. Curam was administered in the drinking water to 24 h before infection. Four mice were gavaged with mixtures of strains J10Sm and AHC-6, and four mice were gavaged with J10Sm and Δ*npsB* (10^5^:10^7^ CFU in CASO broth). Six days post-infection, antibiotic treatment was switched from Curam to rifampicin (0.1 g l^−1^ drinking water) after which mice were housed individually. *K. oxytoca* present in faeces or caecal content were enumerated on CASO-Km, and *K. pneumoniae* J10 densities were determined on CASO-Sm plates. Rif^R^ J10Sm were quantified on CASO-Sm/Rif plates.

### Faecal metabolites

The synthesis of ^15^N-labelled TM and TV used for analyte quantification was described previously^32^. TM and TV were extracted from faeces and quantified by HPLC high-resolution electrospray ionization mass spectrometry as previously described^32^. In brief, faecal pellets were collected daily from each mouse and stored in HPLC glass vials at −80 °C. For extraction, pre-weighed samples were homogenized by vortexing. EtOH-dissolved ^15^N-labelled TM (20 µM) and TV (0.2 µM) were added as internal standards to each sample and vortexed again for 5 min. Samples were evaporated to dryness (10 mbar, 40 °C, 60 min), rehydrated in 20 µl water to maximize signal-to-noise ratio and extracted with 200 µl *n*-butanol by vortexing for 5 min. Extracts were centrifuged in glass vials (15 min, 4,000*g*, 20 °C), the organic phase was filtered (0.2 µm, Nylon) and stored at −20 °C until measurement. For each sample set, the lowest measurable TM concentration was used to define the limit of quantification.

### Bacterial 16S rRNA gene sequencing

For the data shown in Fig. 2 and Extended Data Fig. 2, DNA of 64 murine faecal samples and 16 caecal samples was isolated with the PowerSoil DNA Isolation Kit (Mo Bio Laboratories). These DNA and two control samples (DNA extraction without faeces) were sequenced in two independent Illumina MiSeq, v3, 2x300bp (2 Mio. read package) runs (run 1, caecal; run 2, faecal) by Microsynth AG. For the longitudinal experiment (Fig. 3 and Extended Data Fig. 3), mouse faecal pellets (*n* = 108) and two empty tubes (blank) were sent to Microsynth AG for DNA extraction and Illumina MiSeq, v3, 2x300bp (15 Mio. read package) sequencing (run 3; Fig. 3). For library preparation, dual-indexed universal primers 341F (5′-CCTACGGGNGGCWGCAG-3′) and 802R (5′-GACTACHVGGGTATCTAATCC-3′) were utilized in the two-step PCR amplification of the V3–V4 hypervariable regions of the 16S rRNA gene.

### Data processing, filtering and statistical analysis

Data received from Microsynth AG was demultiplexed and trimmed for Illumina adaptor residuals and locus-specific primers. In total 6,404,412 (run 1), 4,285,688 (run 2) and 15,413,804 (run 3) reads were generated. Data analysis was performed with QIIME2 (2021.4) (ref. ^64^) in Miniconda (4.10.3) environment. To obtain amplicon sequence variants, reads were processed with the Divisive Amplicon Denoising Algorithm (DADA2) (ref. ^65^) with quality score threshold set at 25. Alignments were generated with the q2-dada2 plugin. For sequences obtained in run 1 and run 2, the truncation length was set to 276 for forward and 201 for reverse reads. For run 3, truncation was set to 267 for forward and 181 for reverse reads. Bacterial taxonomy was assigned using RESCRIPt^66^ and a sklearn-based naïve Bayes classifier^67^ trained on the SILVA v138 99% 16S full-length database. The first step filtered out features classified as mitochondria and chloroplasts and features detected in empty/blank samples (*n* = 10 features for run 1 and run 2; *n* = 3 for run 3). Next, samples with reads below 10,000 were excluded for run 1 and run 2, and samples with reads below 23,000 were excluded for run 3 from further analysis. After this second filtering step, 16 of 16 mouse samples (caecal content, run 1), 53 of 60 faecal samples (run 2) and 90 of 108 faecal samples (run 3) remained for analysis. Amplicon sequence variants were used to generate the tree for the phylogenetic diversity analysis with FastTree2 and MAFFT^68,69^. Calculation of alpha-diversity indices (observed features, Shannon and Faith’s phylogenetic diversity), beta-diversity indices (Jaccard distance and unweighted UniFrac) and PCoA utilized the q2-diversity pipeline. To standardize the library size across samples, we normalized pre-processed sequencing data by rarefying to a minimum of 10,000 (runs 1 and 2) and 23,000 (run 3) reads per sample, respectively. Recovery rate of unweighted UniFrac was tracked with q2-longitudinal^70^ with the static point set to day −1 (pre-antibiotic). Statistical test for alpha-diversity indices utilized Kruskal–Wallis test and for beta-diversity calculation pairwise permutational multivariate analysis of variance (PERMANOVA) was used. The *P*-value correction was done using the Benjamin–Hochberg false discovery rate (FDR) correction. Differences in abundance of bacterial taxa was calculated using LEfSe^71^. For visualization, GraphPad Prism (9.2.0) and in-house R scripts (version 3.6.2) were used.

### Sequence analysis of *rpoB*

Amplification of *rpoB* was performed with Phusion-polymerase (New England Biolabs) and genomic DNA (Qiagen DNAeasy Blood & Tissue Kit) or whole cells. Primers rpoB_R4_f (5′-GGATATGATCAACGCCAAGC-3′) and rpoB_4R_r (5′-TCGATAGCAGACAGGTAGTG-3′) generated a 360 bp product (nucleotides (nt) 1,470–1,829) for *E. coli* and *K. pneumoniae*. Sanger sequences were obtained with the same primers. Forward and reverse reads were assembled for each clone, and a 333 bp (nt 1,484–1,816) sequence spanning gene cluster I (nt 1,521–1,611) and cluster II (nt 1,686–1,725) was analysed for mutations.

### Expression constructions

All plasmids and oligonucleotides used for cloning are listed in Supplementary Table 2. *K. oxytoca uvrX* and *E. coli* MG1655 *uvrA* and *mutS* were amplified from genomic DNA with the respective primer pairs 1 + 2, 3 + 4, and 5 + 6 (Supplementary Table 2). Amplified genes were placed under control of the *araBAD* operon promoter in pBAD33, and expression was induced by addition of 0.05% arabinose (Ara).

### TM-induced SOS and mutation in vitro

DNA-damage response was monitored in *E. coli* MG1655 carrying the reporter plasmids pUA66-P_*recA*_-GFP, pUA66-P_*sodA*_-GFP, pUA66-P_*katG*_-GFP and pUA66-P_*soxR*_-GFP described previously^72^. Overnight cultures (ONC) were diluted to OD_600_ = 0.05 in a 24-well plate containing 1 ml LB medium with varying concentrations of TM. Controls included 28.8 nM ciprofloxacin, 0.25 mM paraquat (Sigma-Aldrich) or 0.5% EtOH. OD_600_ and fluorescence (GFP 480/510) of these cultures were recorded in a microplate reader (TECAN GENios Pro v3.40 01/06) for 16 h at 37 °C. *E. coli* MG1655 pUA66 served as autofluorescence control.

For mutagenesis, ONCs of rifampicin-sensitive *E. coli* MG1655 were diluted to OD_600_ = 0.05 in LB medium. Aliquots of 2 ml were incubated (16 h, 180 rpm, 37 °C) in glass eprouvettes with TM or EtOH. Cultures were centrifuged for 5 min at 2,300*g*, and pellets were resuspended in fresh LB medium and incubated for 1 h before plating. To determine the frequency of resistance emergence, undiluted suspensions were plated on LB-Rif agar and serial dilutions were plated on LB agar. Mutation frequencies were expressed as rifampicin-resistant mutants (Rif^R^ CFU ml^−1^) divided by total CFU ml^−1^. The assay was performed five times independently. Mutations in the *rpoB* gene were determined as described above for three colonies per assay (*n* = 15) for each treatment.

### *E. coli* viability assays with Keio collection strains

To assess the impact of TM on viability of *E. coli* JW0001-1 and JW4019-1, bacterial suspensions (5 × 10^7^ CFU ml^−1^) were incubated with 170 µM, 85 µM, 42.5 µM TM or 0.5% EtOH in LB for 2 h at 37 °C under aerobic conditions. Serial dilutions for each treatment were plated to determine CFU. Strains carrying the expression plasmids pUvrA_Ec_, pUvrX_Ko_ and the vector control were grown overnight in the presence of Cm and Ara. ONCs were diluted with Cm/Ara medium to 5 × 10^7^ CFU ml^−1^ and incubated in glass eprouvettes with 85 µM TM or with EtOH for 2 h under aerobic conditions. Serial dilutions were plated to determine CFU. Bacterial viability is expressed as percentage of surviving cells in treatment group compared with the solvent control.

### Complementation of TM-induced mutation in vitro

*E. coli* MG1655 harbouring plasmids P_BAD_-*uvrA* (pUvrA_Ec_), P_BAD_-*uvrX* (pUvrX_Ko_), P_BAD_-*mutS* (pMutS_Ec_) or empty vector were grown in LB medium supplemented with Cm and Ara for 5 h at 37 °C × 180 rpm, then diluted to OD_600_ = 0.05 in LB containing Cm and Ara. Equivalent aliquots were exposed to 42.5 µM TM or EtOH for 16 h at 37 °C × 180 rpm. Serial dilutions were plated with and without rifampicin selection to determine mutation frequencies.

### LC_50_ and mutagenesis assays with human and mouse bacterial isolates

*E. coli* B13, *K. pneumoniae* J10 and *P. aeruginosa* J17 were grown in CASO medium overnight, diluted 1:10 in 5 ml and incubated for 30 min at 37 °C × 180 rpm. Suspensions (OD_600_ = 0.1) were prepared in PBS, then incubated with varying concentrations of TM for 2 h at 37 °C under anaerobic conditions. EtOH was used as solvent control. Viable cells were quantified with one portion of treated cultures by plating serial dilutions on CASO agar. CASO broth was added to the remaining culture to a final volume of 4 ml, and cultures were incubated for 6 h (*E. coli* and *K. pneumoniae*) or 22 h (*P. aeruginosa*) at 37 °C × 180 rpm. The frequencies of RifR emergence were determined by plating aliquots on CASO agar with and without selection. The mutation spectrum obtained in vitro was determined by sequencing the *rpoB* alleles of RifR colonies as described above.

### Statistical analysis

Collection of human samples was randomized and blinded. No other data collection and analysis was performed blind to the conditions of the experiments. No statistical methods were used to pre-determine sample sizes, but our sample sizes are similar to those reported in previous publications^20,72,73^. Mice were randomly assigned to respective treatment groups. No animals or data points were excluded from the analyses. Data collection, statistical analysis and visualization utilized Microsoft Excel (22.03) GraphPad Prism (9.2.0), CorelDRAW 2019 (21.0.0.593) and in-house R scripts (3.6.2). The following packages were used with R (3.6.2): ggplot2 (2.3.5), qiime2R (0.99.6), dplyr (1.0.8), tidyverse (1.3.1) and RStudio (1.2.5033). Shapiro–Wilk normality test was applied to all datasets *n* > 6; for all other sets, we assumed non-normal distribution, but this was not formally tested. Testing for statistical significance always employed two-tailed tests if not stated otherwise.

### Reporting summary

Further information on research design is available in the Nature Research Reporting Summary linked to this article.

## Supplementary information

Supplementary Information Supplementary Tables 1–3 and Figs. 1 and 2.

Reporting Summary

## Data Availability

The datasets generated and analysed during the current study are available in the GenBank repository, BioProject ID PRJNA799913. Source data are provided with this paper.

## Source data

Source Data Fig. 1 Statistical source data.
Source Data Fig. 2 Statistical source data.
Source Data Fig. 3 Statistical source data.
Source Data Fig. 4 Statistical source data.
Source Data Fig. 5 Statistical source data.
Source Data Fig. 6 Statistical source data.
Source Data Extended Data Fig. 1 Statistical source data.
Source Data Extended Data Fig. 2 Statistical source data.
Source Data Extended Data Fig. 3 Statistical source data.
Source Data Extended Data Fig. 4 Statistical source data.
Source Data Extended Data Fig. 5 Statistical source data.
Source Data Extended Data Fig. 6 Statistical source data.

## References

[1] Donia MS (2014). A systematic analysis of biosynthetic gene clusters in the human microbiome reveals a common family of antibiotics. Cell.

[2] Shine EE, Crawford JM (2021). Molecules from the microbiome. Annu. Rev. Biochem..

[3] Kim HU, Blin K, Lee SY, Weber T (2017). Recent development of computational resources for new antibiotics discovery. Curr. Opin. Microbiol..

[4] Medema MH, Fischbach MA (2015). Computational approaches to natural product discovery. Nat. Chem. Biol..

[5] Rutledge PJ, Challis GL (2015). Discovery of microbial natural products by activation of silent biosynthetic gene clusters. Nat. Rev. Microbiol..

[6] Rebuffat S (2022). Ribosomally synthesized peptides, foreground players in microbial interactions: recent developments and unanswered questions. Nat. Prod. Rep..

[7] Walsh CT, Fischbach MA (2010). Natural Products Version 2.0: connecting genes to molecules. J. Am. Chem. Soc..

[8] da Silva RR, Dorrestein PC, Quinn RA (2015). Illuminating the dark matter in metabolomics. Proc. Natl Acad. Sci..

[9] Silpe JE, Balskus EP (2021). Deciphering human microbiota–host chemical interactions. ACS Cent. Sci..

[10] Engevik, M. A. & Versalovic, J. Biochemical features of beneficial microbes: foundations for therapeutic microbiology. *Microbiol. Spectr*. **5**, 10.1128/microbiolspec.BAD-0012-2016 (2017). 10.1128/microbiolspec.bad-0012-2016 PMC5873327 28984235

[11] Vogt SL, Peña-Díaz J, Finlay BB (2015). Chemical communication in the gut: effects of microbiota-generated metabolites on gastrointestinal bacterial pathogens. Anaerobe.

[12] Dornisch E (2017). Biosynthesis of the enterotoxic pyrrolobenzodiazepine natural product tilivalline. Angew. Chem. Int. Ed..

[13] Cosic A (2021). Variation in accessory genes within the *Klebsiella oxytoca* species complex delineates monophyletic members and simplifies coherent genotyping. Front. Microbiol..

[14] Cuénod A (2021). Whole-genome sequence-informed MALDI-TOF MS diagnostics reveal importance of *Klebsiella oxytoca* group in invasive infections: a retrospective clinical study. Genome Med..

[15] Shao Y (2019). Stunted microbiota and opportunistic pathogen colonization in caesarean-section birth. Nature.

[16] Li X (2021). The infant gut resistome associates with *Escherichia coli*, environmental exposures, gut microbiome maturity, and asthma-associated bacterial composition. Cell Host Microbe.

[17] Fanaro S, Chierici R, Guerrini P, Vigi V (2007). Intestinal microflora in early infancy: composition and development. Acta Paediatr..

[18] Taieb, F., Petit, C., Nougayrède, J.-P. & Oswald, E. The enterobacterial genotoxins: cytolethal distending toxin and colibactin. *EcoSal Plus*, **7**, ecosalplus.ESP-0008-2016 (2016). 10.1128/ecosalplus.esp-0008-2016 PMC11575708 27419387

[19] Chen J (2022). A commensal-encoded genotoxin drives restriction of *Vibrio cholerae* colonization and host gut microbiome remodeling. Proc. Natl Acad. Sci. USA.

[20] Unterhauser K (2019). *Klebsiella oxytoca* enterotoxins tilimycin and tilivalline have distinct host DNA-damaging and microtubule-stabilizing activities. Proc. Natl Acad. Sci. USA.

[21] Wernke KM (2020). Structure and bioactivity of colibactin. Bioorg. Med. Chem. Lett..

[22] Högenauer C (2006). *Klebsiella oxytoca* as a causative organism of antibiotic-associated hemorrhagic colitis. N. Engl. J. Med..

[23] Neog N, Phukan U, Puzari M, Sharma M, Chetia P (2021). *Klebsiella oxytoca* and emerging nosocomial infections. Curr. Microbiol..

[24] Paveglio S (2020). Cytotoxin-producing *Klebsiella oxytoca* in the preterm gut and its association with necrotizing enterocolitis. Emerg. Microbes Infect..

[25] Alexander EM (2020). Biosynthesis, mechanism of action, and inhibition of the enterotoxin tilimycin produced by the opportunistic pathogen *Klebsiella oxytoca*. ACS Infect. Dis..

[26] Heilbronner S, Krismer B, Brötz-Oesterhelt H, Peschel A (2021). The microbiome-shaping roles of bacteriocins. Nat. Rev. Microbiol..

[27] Russell AB, Peterson SB, Mougous JD (2014). Type VI secretion system effectors: poisons with a purpose. Nat. Rev. Microbiol..

[28] Ruhe ZC, Low DA, Hayes CS (2020). Polymorphic toxins and their immunity proteins: diversity, evolution, and mechanisms of delivery. Annu. Rev. Microbiol..

[29] Blázquez J, Rodríguez-Beltrán J, Matic I (2018). Antibiotic-induced genetic variation: how it arises and how it can be prevented. Annu. Rev. Microbiol..

[30] Higaki M, Chida T, Takano H, Nakaya R (1990). Cytotoxic component(s) of *Klebsiella oxytoca* on HEp-2 cells. Microbiol. Immunol..

[31] Zollner‐Schwetz I (2008). Role of *Klebsiella oxytoca* in antibiotic‐associated diarrhea. Clin. Infect. Dis..

[32] Glabonjat RA (2021). Simultaneous quantification of enterotoxins tilimycin and tilivalline in biological matrices using HPLC high resolution ESMS2 based on isotopically ^15^N-labeled internal standards. Talanta.

[33] Schneditz G (2014). Enterotoxicity of a nonribosomal peptide causes antibiotic-associated colitis. Proc. Natl Acad. Sci. USA.

[34] Brook I, Pazzaglia G, Coolbaugh JC, Walker RI (1983). In-vivo protection of group A β-haemolytic streptococci from penicillin by β-lactamase-producing *Bacteroides* species. J. Antimicrob. Chemother..

[35] Brook I, Pazzaglia G, Coolbaugh JC, Walker RI (1984). In vivo protection of penicillin-susceptible *Bacteroides melaninogenicus* from penicillin by facultative bacteria which produce beta-lactamase. Can. J. Microbiol..

[36] Vatanen T (2018). The human gut microbiome in early-onset type 1 diabetes from the TEDDY study. Nature.

[37] Dethlefsen L, Relman DA (2011). Incomplete recovery and individualized responses of the human distal gut microbiota to repeated antibiotic perturbation. Proc. Natl Acad. Sci. USA.

[38] Crane JK, Alvarado CL, Sutton MD (2021). Role of the SOS response in the generation of antibiotic resistance in vivo. Antimicrob. Agents Chemother..

[39] Cirz RT (2005). Inhibition of mutation and combating the evolution of antibiotic resistance. PLoS Biol..

[40] Woodford N, Ellington MJ (2007). The emergence of antibiotic resistance by mutation. Clin. Microbiol. Infect..

[41] Baharoglu Z, Mazel D (2011). *Vibrio cholerae* triggers SOS and mutagenesis in response to a wide range of antibiotics: a route towards multiresistance. Antimicrob. Agents Chemother..

[42] Gutierrez A (2013). β-Lactam antibiotics promote bacterial mutagenesis via an RpoS-mediated reduction in replication fidelity. Nat. Commun..

[43] Mathieu A (2016). Discovery and function of a general core hormetic stress response in *Escherichia coli* induced by sublethal concentrations of antibiotics. Cell Rep..

[44] Goodman MF (2016). Better living with hyper-mutation: hypermutation. Environ. Mol. Mutagen..

[45] Cundliffe E, Demain AL (2010). Avoidance of suicide in antibiotic-producing microbes. J. Ind. Microbiol. Biotechnol..

[46] Snitkin ES (2012). Tracking a hospital outbreak of carbapenem-resistant *Klebsiella pneumoniae* with whole-genome sequencing. Sci. Transl. Med..

[47] Boucher HW (2009). Bad Bugs, No Drugs: No ESKAPE! An update from the Infectious Diseases Society of America. Clin. Infect. Dis..

[48] Iredell J, Brown J, Tagg K (2016). Antibiotic resistance in Enterobacteriaceae: mechanisms and clinical implications. BMJ.

[49] Falagas ME, Kastoris AC, Kapaskelis AM, Karageorgopoulos DE (2010). Fosfomycin for the treatment of multidrug-resistant, including extended-spectrum β-lactamase producing, Enterobacteriaceae infections: a systematic review. Lancet Infect. Dis..

[50] Osbelt L (2021). *Klebsiella oxytoca* causes colonization resistance against multidrug-resistant *K. pneumoniae* in the gut via cooperative carbohydrate competition. Cell Host Microbe.

[51] Beaugerie L (2003). *Klebsiella oxytoca* as an agent of antibiotic-associated hemorrhagic colitis. Clin. Gastroenterol. Hepatol..

[52] Greimel TM (2022). Toxin-producing *Klebsiella oxytoca* in healthy infants: commensal or pathobiont?. J. Pediatr. Gastroenterol. Nutr..

[53] Bäckhed F (2015). Dynamics and stabilization of the human gut microbiome during the first year of life. Cell Host Microbe.

[54] Yatsunenko T (2012). Human gut microbiome viewed across age and geography. Nature.

[55] Subramanian S (2014). Persistent gut microbiota immaturity in malnourished Bangladeshi children. Nature.

[56] Bokulich NA (2016). Antibiotics, birth mode, and diet shape microbiome maturation during early life. Sci. Transl. Med..

[57] Yassour M (2016). Natural history of the infant gut microbiome and impact of antibiotic treatment on bacterial strain diversity and stability. Sci. Transl. Med..

[58] Vatanen T (2016). Variation in microbiome LPS immunogenicity contributes to autoimmunity in humans. Cell.

[59] Olin A (2018). Stereotypic immune system development in newborn children. Cell.

[60] Arrieta M-C (2015). Early infancy microbial and metabolic alterations affect risk of childhood asthma. Sci. Transl. Med..

[61] Stokholm J (2018). Maturation of the gut microbiome and risk of asthma in childhood. Nat. Commun..

[62] Morley VJ, Woods RJ, Read AF (2019). Bystander selection for antimicrobial resistance: implications for patient health. Trends Microbiol..

[63] Geboes K (2000). A reproducible grading scale for histological assessment of inflammation in ulcerative colitis. Gut.

[64] Bolyen E (2019). Reproducible, interactive, scalable and extensible microbiome data science using QIIME 2. Nat. Biotechnol..

[65] Callahan BJ (2016). DADA2: high-resolution sample inference from Illumina amplicon data. Nat. Methods.

[66] Ii MSR (2021). RESCRIPt: reproducible sequence taxonomy reference database management. PLoS Comput. Biol..

[67] Pedregosa F (2011). Scikit-learn: machine learning in Python. J. Mach. Learn. Res..

[68] Price MN, Dehal PS, Arkin AP (2010). FastTree 2—approximately maximum-likelihood trees for large alignments. PLoS ONE.

[69] Katoh K, Standley DM (2013). MAFFT multiple sequence alignment software version 7: improvements in performance and usability. Mol. Biol. Evol..

[70] Bokulich NA (2018). q2-longitudinal: longitudinal and paired-sample analyses of microbiome data. mSystems.

[71] Segata N (2011). Metagenomic biomarker discovery and explanation. Genome Biol..

[72] Giroux X, Su W-L, Bredeche M-F, Matic I (2017). Maladaptive DNA repair is the ultimate contributor to the death of trimethoprim-treated cells under aerobic and anaerobic conditions. Proc. Natl Acad. Sci. USA.

[73] Kienesberger S (2016). Gastric *Helicobacter pylori* infection affects local and distant microbial populations and host responses. Cell Rep..

