# Supplementary material for: Enterotoxin tilimycin from gut-resident Klebsiella promotes mutational evolution and antibiotic resistance in mice
Source: Nat Microbiol. 2022 Oct 26;7(11):1834–48. doi: 10.1038/s41564-022-01260-3 (PMC9613472; doi:10.1038/s41564-022-01260-3)
Supplement: Supplementary file 1 — Supplementary Tables 1–3 and Figs. 1 and 2. [file 41564_2022_1260_MOESM1_ESM.pdf]

nature microbiology

Article

https://doi.org/10.1038/s41564-022-01260-3

Enterotoxin tilimycin from gut-resident *Klebsiella* promotes mutational evolution and antibiotic resistance in mice

In the format provided by the authors and unedited

Supplementary Tables

Table S1. Spectrum of *E. coli rpoB* mutations obtained in vivo and in vitro.

sequence context			Number (%)				
			in vivo (mouse)		*in vitro*		
			Toxin+	Toxin-	Toxin+	Toxin-	CIP
nt* in *rpoB*	**5'- 3'**	**Mutation**	**189**	**8**	**12**	**5**	**5**
1532	GC**T**GT	1 T A→CG	1 (0.5)	-	-	-	-
1533-1538	(<u>GTCTCA</u>)	(<u>Deletion</u>)	2 (1.1)	-	-	-	-
1534	TG**T**CT	TA→CG	2 (1.1)	2 (25.0)	-	-	-
1535	GT**C**TC	CG→TA	5 (2.6)	1 (12.5)	-	-	-
1537	CT**C**AG	CG→AT	-	-	-	1 (20.0)	-
1546	TG**G**AC	GC→AT	136 (72.0)	-	10 (83.3)	1 (20.0)	-
1547	GG**A**CC	AT→GC	27 (14.3)	2 (25.0)	-	-	-
1574	TA**C**GC	CG→GC	1 (0.5)	-	-	-	-
1576	CG**C**AC	CG→TA	3 (1.6)	1 (12.5)	-	-	-
1585	GT**C**GT	CG→TA	1 (0.5)	-	-	-	-
1586	TC**G**TA	GC→AT	2 (1.1)	-	-	-	-
1592	CT**C**CG	CG→TA	6 (3.2)	-	2 (16.7)	1 (20.0)	-
1691	CC**C**TG	CG→TA	3 (1.6)	1 (12.5)	-	2 (40.0)	1 (20.0)
1714	TG**A**TC	AT→TA	-	-	-	-	4 (80.0)
1715	GA**T**CA	TA→CG	-	1 (12.5)	-	-	-

nt, nucleotide; **bold,** base substitution; CIP, ciprofloxacin; *The A of the ATG initiation codon of *rpoB* is nucleotide 1 according to GenBank accession CP032679.1, region 4,171,142..4,175,170, locus tag b3987. The sequence of *rpoB* of mouse isolate B13 is identical to MG1655 in the region analyzed.

Table S2. Strains, Plasmids, and Oligonucleotides used for cloning.

Strain	Genotype/Description	Reference
Klebsiella oxytoca AHC-6	human AAHC isolate 04/1 O, AmpR, Toxin+, ST 1	[1]
K. oxytoca gcvA::Km **(AHC-6)**	AHC-6 with *gcvA*::Tn5-*aphA*, KmR, Toxin+	[2]
K. oxytoca Mut89 **(Δ*npsB*)**	AHC-6 with *npsB*::Tn5-*aphA*, KmR, Toxin-	[2]
Escherichia coli MG1655	K-12	[3]
E. coli B13	mouse isolate, AmpR, CuramR, MIC_{TM} >340 µM	this study
E. coli B13Sm	AmpR, CuramR, SmR	this study
E. coli JW0001-1	Δ*thrA722::kan,* KmR	[4]
E. coli JW4019-2	Δ*uvrA753::kan*, KmR	[4]
K. pneumoniae J10	human isolate, AmpR, MIC_{TM} >340 µM, ST closest match: 2606*, MLST alleles: 79-16-21-27-44-(24)*-67	this study
K. pneumoniae J10Sm	AmpR, SmR	this study
Pseudomonas aeruginosa J17	human isolate, CuramR, PipR, TzpR, CazR, AztR, MIC_{TM} >340 µM	this study

Plasmid	Description	Reference
pNpsB	CmR, pACYC184 with *tetAR* promoter fused to *npsB*	[2]
pUA66	KmR	[5]
pUA66-P_{recA}-GFP	pUA66 *E. coli recA* promoter fused to *gfpmut2*	[5]
pUA66-P_{sodA}-GFP	pUA66 *E. coli sodA* promoter fused to *gfpmut2*	[5]
pUA66-P_{katG}-GFP	pUA66 *E. coli katG* promoter fused to *gfpmut2*	[5]
pUA66-P_{soxS}-GFP	pUA66 *E. coli soxS* promoter fused to *gfpmut2*	[5]
pBAD33	CmR, *araC*, *araBAD* promoter	[6]
$pUvrA_{Ec}$	pBAD with *araBAD* promoter fused to *E. coli uvrA*	this study
$pUvrX_{Ko}$	pBAD with *araBAD* promoter fused to *K. oxytoca uvrX*	this study
$pMutS_{Ec}$	pBAD with *araBAD* promoter fused to *E. coli mutS*	this study

# Oligonucleotide	**Sequence 5'-3'	***Reference
1 SacI_RBS_uvrX_fw	attc**gagctc**gcaggaggaattcaccatgaaaacccatgacgctataaaaatc	HG425356.1
2 SphI_uvrX_long_rev	agctt**gcatgc**ctattgccagtctctactggctg	HG425356.1
3 SacI_RBS_uvrA chrom_fw	at**gagctc**gcaggaggaattcaccatggataagatcgaagttcgg	CP032679.1
4 SphI_Ec-uvrA_rev	agctt**gcatgc**ttacagcatcggcttaaggaagc	CP032679.1
5 SacI_RBS_mutS_fw	attc**gagctc**gcaggaggaattcaccatgagtgcaatagaaaatttcg	CP032679.1
6 SphI_mutS_rev	agctt**gcatgc**cttacaccaggctcttcaagcg	CP032679.1

Resistances (R): Ampicillin (AmpR), Kanamycin (KmR), Amoxicillin/Clavulanic acid (CuramR), Streptomycin (SmR), Piperacilin (PipR), Piperacillin/Tazobactam (TtzpR), Ceftazidim (CazR), Aztreonam (AtzR); Minimal inhibitory concentration of tilimycin (MIC_{TM}); Sequence Type (ST); Multilocus sequence type (MLST) alleles; *not typable, closest match for *infB* according to allelic profile is allele 24; order allelic profile: *rpoB-gapA-mdh-pgi-phoE-infB-tonB*, **Enzyme restriction sites are in **bold** and start codons are underlined. ***Accession number, *til*-gene cluster of *K. oxytoca* AHC-6 (isolate 04/1 O) and *E. coli* MG1655

Table S3. ^{1}H NMR (500 MHz, MeOD-d_4) of the *trans*- and the *cis*-isomer of tilimycin.

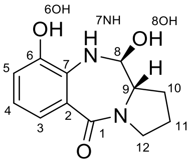

trans-Tilimycin

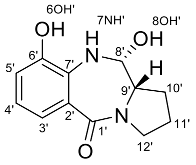

cis-Tilimycin

Atom #	δ_H, mult., *J* (Hz)	Atom #	δ_H, mult., *J* (Hz)
1	—	1’	—
2	—	2’	—
3	7.11, dd (7.5, 1.7)	3’	7.31, dd (8.2, 1.5)
4	6.87, t (7.7)	4’	6.59–6.56, m
5	6.91, dd (7.9, 1.7)	5’	6.81, dd (7.6, 1.5)
6	—	6’	—
7	—	7’	—
8	4.54, d (8.8)	8’	4.75, s
9	3.58–3.47, m	9’	3.83–3.77, m
10	2.21–1.97, m	10’	2.38–2.26, m<br>2.21–1.97, m
11	2.21–1.97, m	11’	2.21–1.97, m<br>1.88–1.77, m
12	3.75–3.65, m<br>3.58–3.47, m	12’	3.75–3.65, m

Supplementary Figures

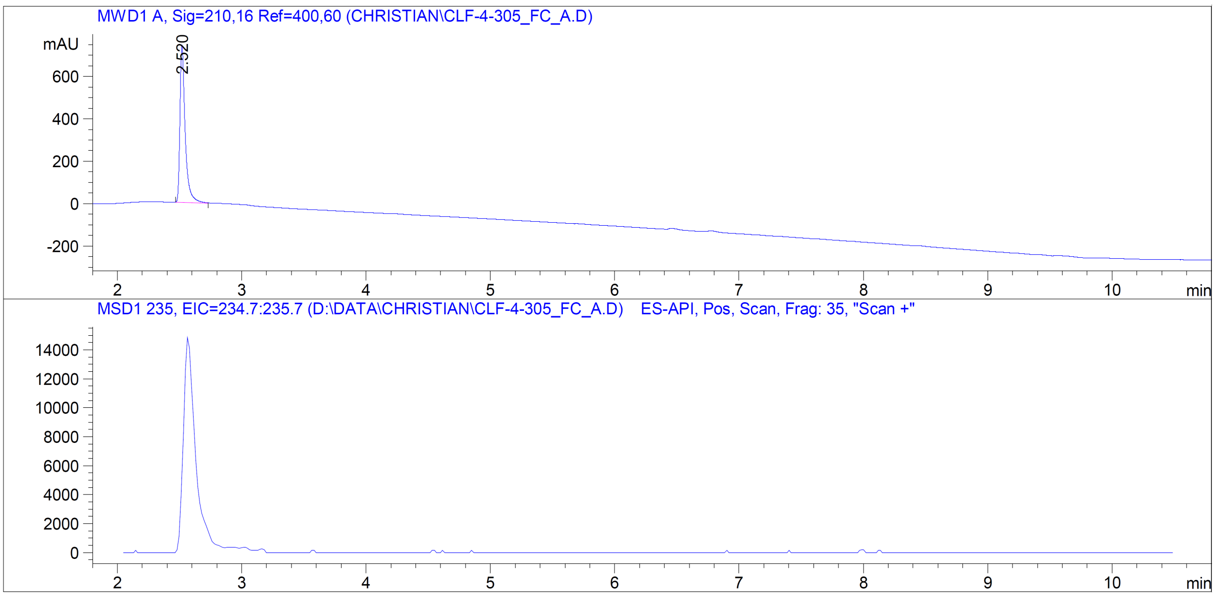

Figure S1. HPLC purity after prepHPLC chromatography.

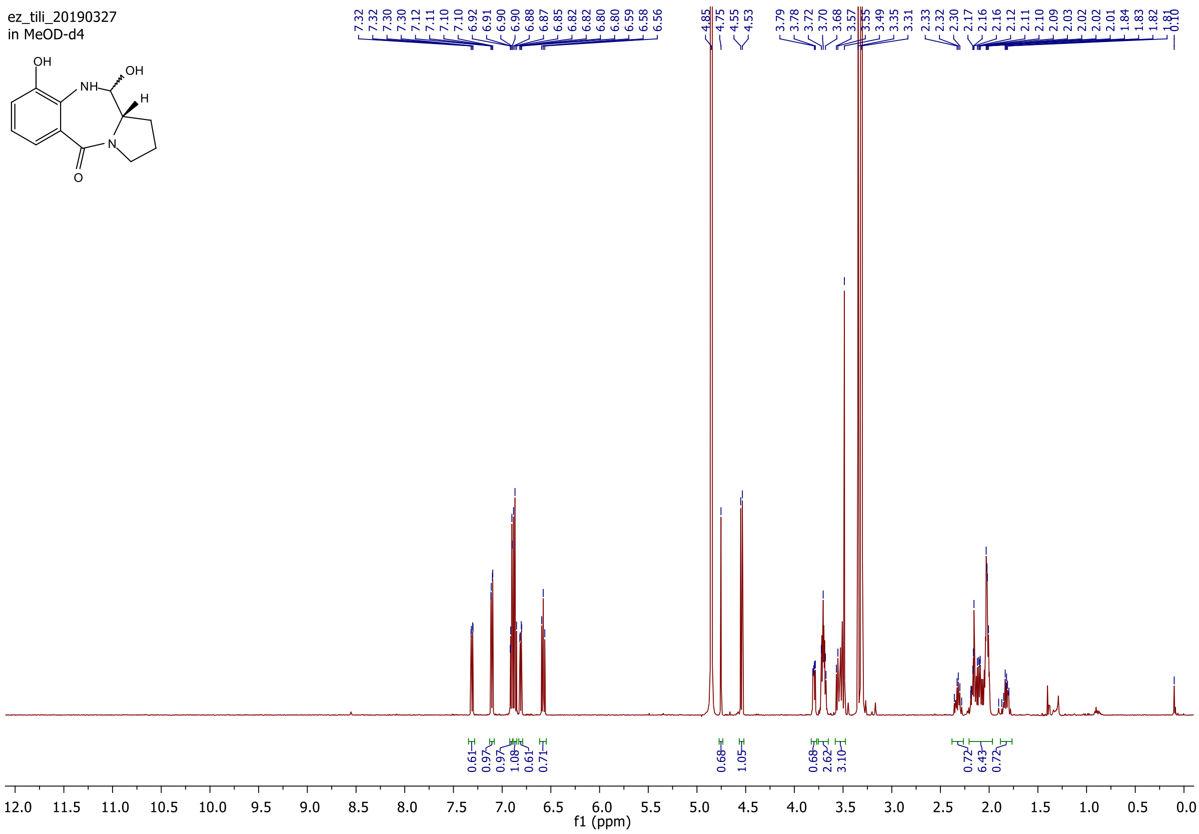

Figure S2. HPLC-HR-ESMS² spectra after flash column.

Supplementary References

1. Joainig, M. M. *et al.* Cytotoxic Effects of *Klebsiella oxytoca* Strains Isolated from Patients with Antibiotic-Associated Hemorrhagic Colitis or Other Diseases Caused by Infections and from Healthy Subjects. *J. Clin. Microbiol.* **48**, 817–824 (2010).
2. Schneditz, G. *et al.* Enterotoxicity of a nonribosomal peptide causes antibiotic-associated colitis. *Proc. Natl. Acad. Sci.* **111**, 13181–13186 (2014).
3. Bachmann, B., J. Derivations and genotypes of some mutant derivatives of *Escherichia coli* K-12. In: *Escherichia coli* and *Salmonella typhimurium* Cellular and Molecular Biology. in *Escherichia coli and Salmonella: cellular and molecular biology* (eds. Neidhardt, F. C. & Curtiss, R.) (ASM Press, 1996).
4. Baba, T. *et al.* Construction of *Escherichia coli* K-12 in-frame, single-gene knockout mutants: the Keio collection. *Mol. Syst. Biol.* **2**, (2006).
5. Giroux, X., Su, W.-L., Bredeche, M.-F. & Matic, I. Maladaptive DNA repair is the ultimate contributor to the death of trimethoprim-treated cells under aerobic and anaerobic conditions. *Proc. Natl. Acad. Sci.* **114**, 11512–11517 (2017).
6. Guzman, L. M., Belin, D., Carson, M. J. & Beckwith, J. Tight regulation, modulation, and high-level expression by vectors containing the arabinose PBAD promoter. *J. Bacteriol.* **177**, 4121–4130 (1995).
