# Supplementary material for: Enterotoxin tilimycin from gut-resident Klebsiella promotes mutational evolution and antibiotic resistance in mice
Source: Nat Microbiol. 2022 Oct 26;7(11):1834–48. doi: 10.1038/s41564-022-01260-3 (PMC9613472; doi:10.1038/s41564-022-01260-3)
Supplement: Supplementary file 2 — Reporting Summary [file 41564_2022_1260_MOESM2_ESM.pdf]

nature portfolio

Corresponding author(s): Ellen L. Zechner

Last updated by author(s): Sep 16, 2022

# Reporting Summary

Nature Portfolio wishes to improve the reproducibility of the work that we publish. This form provides structure for consistency and transparency in reporting. For further information on Nature Portfolio policies, see our Editorial Policies and the Editorial Policy Checklist.

## Statistics

For all statistical analyses, confirm that the following items are present in the figure legend, table legend, main text, or Methods section.

| n/a | Confirmed | |
|---|---|---|
| ☐ | ☒ | The exact sample size ($n$) for each experimental group/condition, given as a discrete number and unit of measurement |
| ☐ | ☒ | A statement on whether measurements were taken from distinct samples or whether the same sample was measured repeatedly |
| ☐ | ☒ | The statistical test(s) used AND whether they are one- or two-sided<br>*Only common tests should be described solely by name; describe more complex techniques in the Methods section.* |
| ☒ | ☐ | A description of all covariates tested |
| ☐ | ☒ | A description of any assumptions or corrections, such as tests of normality and adjustment for multiple comparisons |
| ☐ | ☒ | A full description of the statistical parameters including central tendency (e.g. means) or other basic estimates (e.g. regression coefficient) AND variation (e.g. standard deviation) or associated estimates of uncertainty (e.g. confidence intervals) |
| ☐ | ☒ | For null hypothesis testing, the test statistic (e.g. $F$, $t$, $r$) with confidence intervals, effect sizes, degrees of freedom and $P$ value noted<br>*Give P values as exact values whenever suitable.* |
| ☒ | ☐ | For Bayesian analysis, information on the choice of priors and Markov chain Monte Carlo settings |
| ☒ | ☐ | For hierarchical and complex designs, identification of the appropriate level for tests and full reporting of outcomes |
| ☒ | ☐ | Estimates of effect sizes (e.g. Cohen's $d$, Pearson's $r$), indicating how they were calculated |

*Our web collection on statistics for biologists contains articles on many of the points above.*

## Software and code

Policy information about availability of computer code

| | |
|---|---|
| Data collection | TECAN GENios Pro v3.40 01/06, software XFluor4 v4.15, MARS (v4.00 R2) |
| Data analysis | GraphPad Prism (9.2.0); CorelDRAW 2019 (21.0.0.593); QIIME2(2021.4); Miniconda (4.10.3); DADA2 (1.18); FastTree2 (2.1.11); MAFFT (7.477); RESCRIPT (2021.11.0); LEFSE (1.1.2); R (3.6.2) with packages ggplot2 (3.3.5) qiime2R (0.99.6) dplyr (1.0.8) tidyverse (1.3.1); Microsoft® Excel® for Microsoft 365 MSO (Version 2203); R studio (1.2.5033)<br>Key software and algorithms are also mentioned and cited in the methods section. |

For manuscripts utilizing custom algorithms or software that are central to the research but not yet described in published literature, software must be made available to editors and reviewers. We strongly encourage code deposition in a community repository (e.g. GitHub). See the Nature Portfolio guidelines for submitting code & software for further information.

## Data

Policy information about availability of data

All manuscripts must include a data availability statement. This statement should provide the following information, where applicable:

- Accession codes, unique identifiers, or web links for publicly available datasets
- A description of any restrictions on data availability
- For clinical datasets or third party data, please ensure that the statement adheres to our policy

The datasets generated and analysed during the current study are available in the GenBank repository, BioProject ID PRJNA799913.
https://www.ncbi.nlm.nih.gov/bioproject/PRJNA799913

# Field-specific reporting

Please select the one below that is the best fit for your research. If you are not sure, read the appropriate sections before making your selection.

- [x] Life sciences
- [ ] Behavioural & social sciences
- [ ] Ecological, evolutionary & environmental sciences

For a reference copy of the document with all sections, see nature.com/documents/nr-reporting-summary-flat.pdf

# Life sciences study design

All studies must disclose on these points even when the disclosure is negative.

| | |
|---|---|
| Sample size | No statistical methods were used to pre-determine sample sizes. For animal studies, sample size was chosen according to the 3R principle to reduce the number of animals. In addition, our sample sizes were chosen based on those reported in previous publications [Kienesberger, S. et al. Gastric Helicobacter pylori Infection Affects Local and Distant Microbial Populations and Host Responses. Cell Rep. 14, 1395–1407 (2016); Unterhauser, K. et al. Klebsiella oxytoca enterotoxins tilimycin and tilivalline have distinct host DNA-damaging and microtubule-stabilizing activities. Proc Natl Acad Sci USA 116, 3774–3783 (2019)]. Experiments were designed and performed sequentially to allow adjustment of sample sizes in follow-up experiments. Growth curves, fluorescence measurements and toxicity assays were always performed with n>3. Samples sizes were chosen based on previous publications using these reagents [Giroux, X., et al. Maladaptive DNA repair is the ultimate contributor to the death of trimethoprim-treated cells under aerobic and anaerobic conditions. Proc. Natl. Acad. Sci. 114, 11512–11517 (2017)].<br>Sample sizes are indicated in all figure legends. We always assumed non-parametric data to keep data analysis stringent. Shapiro-Wilk normality test was applied to all datasets n>6. For all other sets we assumed non-normal distribution but this was not formally tested. Testing for statistical significance always employed two-tailed tests if not stated otherwise. |
| Data exclusions | No data was excluded |
| Replication | For 16S rRNA data we report the results of two independent animal experiments with n=4-6 mice per group (Figures 2 and 3). Presented results verify reproducibility of the experiments.<br>Figure 4 presents a summary of in vivo data from 3 independent experiments (n=3-4 mice per group). Each experiment was a successful replication. Data was combined to achieve a larger number of samples for powerful statistical analysis of highly variable mutation frequencies.<br>Data presented in Figure 5 was collected from one mouse experiment with n=4 mice per group. Due to the clear outcome we chose to maximize animal welfare (3R) and omit replication.<br>All in vitro assays and metabolite quantification in collected mouse stools were performed in at least 3 biological/independent replicates. All replication attempts were successful. |
| Randomization | Human stool samples were randomized and blinded.<br>For animal studies all mice were randomly assigned to groups. To protect animal welfare and reduce costs we co-housed mice in groups of 2 and controlled for covariances (ie. animal handling, cage effects, contaminations, sample size,...) during data analyses. |
| Blinding | Collection of human samples was randomized and blinded. All other data collection and analysis were not performed blind to the conditions of the experiments. This is common for the type of experiments conducted where knowledge of strain identity/treatment is needed for analysis and sample processing. |

# Reporting for specific materials, systems and methods

We require information from authors about some types of materials, experimental systems and methods used in many studies. Here, indicate whether each material, system or method listed is relevant to your study. If you are not sure if a list item applies to your research, read the appropriate section before selecting a response.

## Materials & experimental systems

| n/a | Involved in the study | |
|---|---|---|
| x | | Antibodies |
| x | | Eukaryotic cell lines |
| x | | Palaeontology and archaeology |
| | x | Animals and other organisms |
| | x | Human research participants |
| x | | Clinical data |
| x | | Dual use research of concern |

## Methods

| n/a | Involved in the study | |
|---|---|---|
| x | | ChIP-seq |
| x | | Flow cytometry |
| x | | MRI-based neuroimaging |

## Animals and other organisms

Policy information about studies involving animals; ARRIVE guidelines recommended for reporting animal research

| | |
|---|---|
| Laboratory animals | Mus musculus, female, C57BL/6NRj and C57Bl/6J, 7-8 weeks |
| Wild animals | This study did not involve wild animals |
| Field-collected samples | This study did not involve samples collected from the field. |
| Ethics oversight | All mouse studies were performed in accordance with the Commission for Animal Experiments of the Austrian Ministry of Science (GZ BMWFW-66.007/0002-WF/V/3b/2017 and BMWFW-39/12175ex2017/18) and the local ethics committee of the University of Graz. Mice were housed in specific pathogen-free conditions in individually ventilated cages and maintained on a 12 h light/dark cycle. Food and water were offered ad libitum. Mice were monitored daily to record their weight and stress levels and killed at the study endpoint with isoflurane and cervical dislocation. |

Note that full information on the approval of the study protocol must also be provided in the manuscript.

## Human research participants

Policy information about studies involving human research participants

| | |
|---|---|
| Population characteristics | Population characteristics were not important for the type of analysis we performed on human stool samples and thus, no data was collected. |
| Recruitment | Nine stool donors (male and female) were recruited at the Medical University of Graz. Healthy donors were selected. Subjects who received antibiotics within 8 weeks prior to sampling were excluded. Samples were collected by donors and pseudonymized by Dr.med Christoph Högenauer. Sampling and selection biases are not expected due to the nature of the experiments performed. |
| Ethics oversight | Informed written consent was obtained from donors and stool samples were collected and pseudonymized by Dr. C. Högenauer as approved by the Institutional Review Board of the Medical University of Graz (17-199 ex 05/06). No personal data was collected from human stool donors for this study. |

Note that full information on the approval of the study protocol must also be provided in the manuscript.
